# A signal-responsive cooperative transcription factor network determines alveolar macrophage identity

**DOI:** 10.1084/jem.20242085

**Published:** 2026-06-02

**Authors:** Jorge Mínguez-Martínez, Jesús Porcuna, Laura Casablanca, Manuel J. Gómez, Hooman Aghelpasand, Pontus Höjer, Belén García-Fojeda, Vanessa Núñez, Carlos Torroja, Severin Mühleder, Charlotte Gustafsson, Thierry Fischer, Rui Benedito, Cristina Casals, Robert Månsson, Fátima Sánchez-Cabo, Mercedes Ricote

**Affiliations:** 1 Cardiovascular Regeneration Program, Centro Nacional de Investigaciones Cardiovasculares (CNIC), Madrid, Spain; 2 Department of Immunology and Oncology, Centro Nacional de Biotecnología (CNB), Consejo Superior de Investigaciones Científicas (CSIC), Madrid, Spain; 3 Bioinformatics Unit, Centro Nacional de Investigaciones Cardiovasculares (CNIC), Madrid, Spain; 4 Department of Gene Technology, National Genomics Infrastructure, Science for Life Laboratory, KTH - Royal Institute of Technology, Stockholm, Sweden; 5 Department of Biochemistry and Molecular Biology, Complutense University of Madrid, Madrid, Spain; 6 Division of Clinical Immunology, Department of Laboratory Medicine, Karolinska Institutet, Stockholm, Sweden; 7 Department of Clinical Immunology and Transfusion Medicine, Karolinska University Hospital, Stockholm, Sweden

## Abstract

Tissue-resident macrophages receive signals from their microenvironment that coordinate the activity of transcription factors (TFs) to establish distinct transcriptional profiles and identities. However, the molecular mechanisms whereby interactions with other cells of the niche imprint a distinct macrophage identity remain poorly understood. Here, we report that retinoid X receptors (RXRs) determine the differentiation and identity of alveolar macrophages (AM) by regulating chromatin accessibility and transcriptional activity of AM-core and function genes, enabling PPARγ-dependent programs. AM differentiation and maintenance *in vivo* require RXR upregulation in response to tissue-derived δ-like canonical Notch ligand 4 (DLL4), GM-CSF, and TGF β (TGFβ). Interplay among these signals leads to cooperation between RXRα, RBPJ, STAT5, and SMAD4 for the transcriptional and epigenetic regulation of key AM-core genes. These results underscore the role of RXRs as key TFs that cooperate with other regulatory elements to establish the AM population and determine AM identity.

## Teaser

Tissue-resident macrophages acquire distinct identities shaped by local signals. Mínguez-Martínez et al. show that RXRs drive alveolar macrophage differentiation by regulating chromatin and gene expression in cooperation with other regulatory elements. DLL4, GM-CSF, and TGFβ coordinate transcriptional networks that, together with the RXR–PPARγ axis, establish and maintain alveolar macrophage population and identity.

## Graphical Abstract

**Figure:**
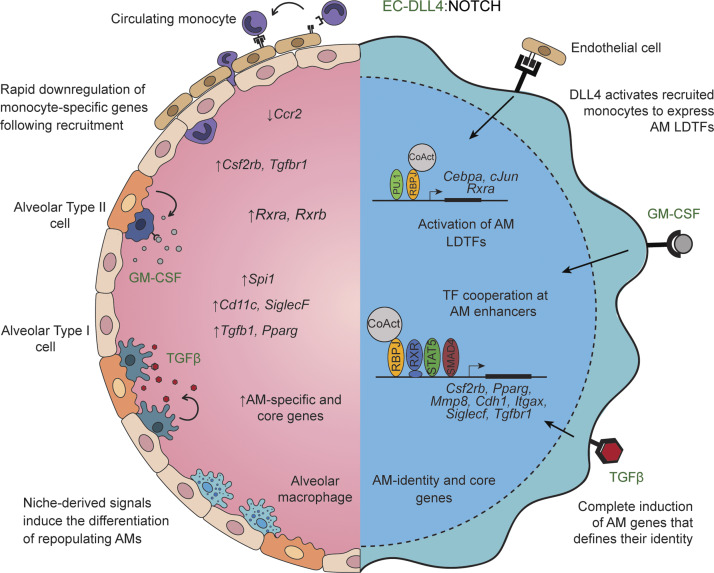

## Introduction

Tissue-resident macrophages (TRMs) are specialized immune cells that reside in all tissues, play crucial roles in pathogen defense, and perform tissue-specific functions in their niche to maintain tissue homeostasis (Hashimoto et al., 2013). TRMs are derived from embryonic precursors and are known to self-renew locally in some tissues, maintaining a stable population throughout life (Mass et al., 2016; Epelman et al., 2014). TRM identity is dictated by instructive niche signals (Porcuna et al., 2020) that direct precursor cells to adopt finely tuned tissue-specific phenotypes. These signals prompt an orchestrated time-dependent expression of transcription factors (TFs) that TRMs require for their correct differentiation and maturation. The core of initial macrophage lineage-determining TFs (LDTFs) is composed of the myeloid pioneer TF PU.1, which is expressed by all TRMs, together with CCAAT/enhancer-binding protein α, MAF BZIP TF (c-MAF), and zinc finger E-box–binding homeobox 2 (ZEB2). Although these TFs work together to shape shared TRM functions (Bleriot et al., 2020), niche-specific signals induce a second group of TFs, known as signal-dependent TFs (SDTFs), that shape distinctive transcriptional programs and epigenetic landscapes (Porcuna et al., 2020; Bleriot et al., 2020; Lavin et al., 2014). SDTFs furthermore trigger the induction of specific functional modules in each subgroup of TRMs, allowing them to establish their identity and perform their tissue-specific functions (T'Jonck et al., 2018).

Alveolar macrophages (AMs) are TRMs residing in lung alveoli. They participate in airway pathogen clearance and the maintenance of correct levels of pulmonary cholesterol and surfactant (Dorr et al., 2022; Minutti et al., 2016). The diversity of lung macrophages results from their remarkable plasticity and their ability to respond to local environmental cues (Bain and MacDonald, 2022; Guilliams et al., 2013). The critical instructive signals that shape AM function, differentiation, and identity include cytokines, metabolites, surfactant proteins, and elevated oxygen levels in the alveolar space (Da Costa Loureiro et al., 2020; Izquierdo et al., 2018). Mouse AMs originate from myeloid precursors during embryonic development (Cohen et al., 2018; Gomez Perdiguero et al., 2015; Tan and Krasnow, 2016). Although AMs are able to self-renew, cell fate mapping studies reveal that a substantial proportion of adult AMs are descended from bone marrow (BM)-derived monocytes (Gomez Perdiguero et al., 2015; Joshi et al., 2018; Liu et al., 2019). AM identity and maturation are instructed by niche-derived CSF 2 (Csf2, GM-CSF) secreted by lung alveolar type II cells (ATIIs) (Gschwend et al., 2021) and autocrine-produced TGF β (TGFβ) (Yu et al., 2017). One of the key TFs for correct AM differentiation is the nuclear receptor PPARγ, which is required for the acquisition of the characteristic AM gene signature, including lipid metabolism-related genes (Porcuna et al., 2020; Schneider et al., 2014). Despite these advances, it remains largely unknown how the regulatory relationships between AM precursors and their niche lead to the induction of specific SDTFs and ultimately to AM differentiation.

The nuclear receptor superfamily comprises several TFs that act as transcriptional regulators upon ligand activation. Nuclear receptors are highly expressed in TRMs, and their tissue-dependent expression has been functionally validated in several studies (Porcuna et al., 2020). Retinoid X receptors (RXRs) form a distinctive subgroup within the nuclear receptor superfamily and can form homo- and heterodimers with other nuclear receptors (Rőszer et al., 2013). RXRs can be activated by vitamin A derivatives, such as 9-cis retinoic acid and dietary lipids. Hematopoietic cells, including tissue macrophages, express RXRα (NR2B1) and RXRβ (NR2B2) but not RXRγ (NR2B3) (Casanova-Acebes et al., 2020). Elevated expression of these receptors is a shared feature of the macrophage lineage (Mass et al., 2016). RXRs regulate a wide range of biological processes in macrophages, including cell differentiation, proliferation, survival, immune responses, and metabolism (Rőszer et al., 2013). RXR signaling ensures correct bone homeostasis and remodeling by maintaining osteoclast differentiation, proliferation, and activity (Menendez-Gutierrez et al., 2015; Menendez-Gutierrez and Ricote, 2017). RXRs also ensure the neonatal expansion of peritoneal macrophages (PMs) by modulating chromatin accessibility and regulate survival and lipid homeostasis in adult cells (Casanova-Acebes et al., 2020).

In the present study, we show that targeted RXR deficiency impairs the maturation of precursors into fully differentiated and functional AMs. Genome-wide analysis revealed that RXRα acts as a master macrophage LDTF, controlling AM identity through the expression of AM-specific TFs. We also demonstrate that the maintenance of adult AM populations and the RXR-controlled AM core program require signaling via δ-like canonical Notch ligand 4 (DLL4) expressed on endothelial cells of the pulmonary microvasculature. RXR expression was induced in AM precursors upon exposure to the alveolar lung-derived signals DLL4, GM-CSF, and TGFβ *in vitro*. Chromatin immunoprecipitation (ChIP) analysis revealed that RXRs establish the core program that defines AM identity through orchestrated cooperation with the TFs RBPJ, STAT5, and SMAD4, activated downstream of the identified niche signals.

## Results

### Deletion of RXRα and RXRβ reduces AM numbers and impairs AM maturation and function

**Figure S1.**
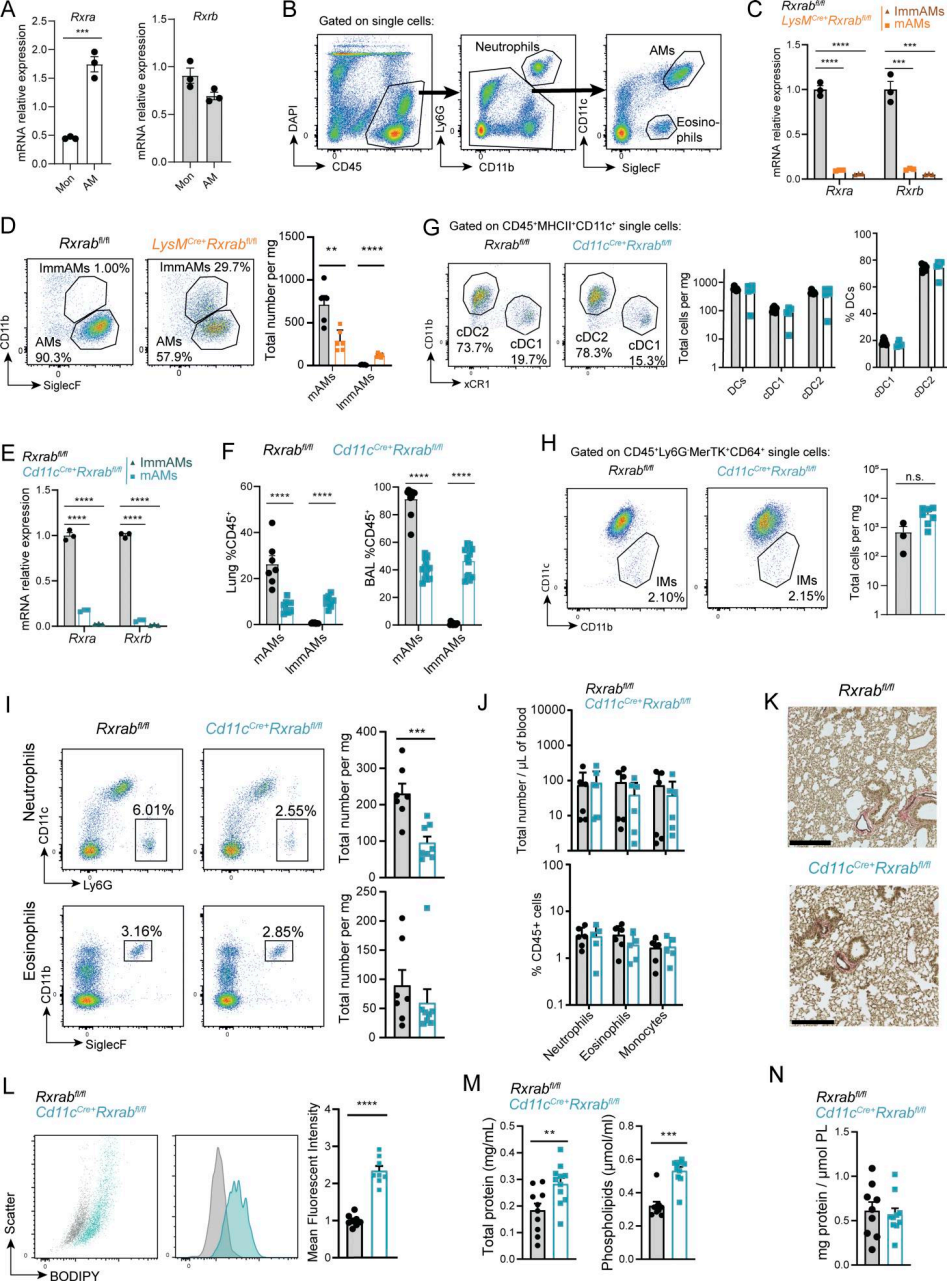
**Deletion of RXRα and RXRβ signaling alters AM phenotype. (A)** mRNA relative expression of *Rxra* and *Rxrb* in monocytes (Mon) and AMs, *n* = 3; each sample was pooled from four mice. **(B)** Gating strategy used to analyze AMs. **(C)** mRNA relative expression analysis of *Rxra* and *Rxrb* deletion in mAMs and ImmAMs from 9-wk-old *Rxrab*^*fl/fl*^ and *LysM*^*Cre+*^*Rxrab*^*fl/fl*^ mice, *n* = 3 per genotype. **(D)** Representative flow cytometry dot plots showing CD11b and SiglecF-gated AMs in whole digested lung from 9-wk-old *Rxrab*^*fl/fl*^ and *LysM*^*Cre+*^*Rxrab*^*fl/fl*^ mice; the graph shows total numbers of mAMs and ImmAMs per mg of tissue. *n* = 5–6 per genotype; data are from two pooled independent experiments. **(E)** mRNA relative expression analysis of *Rxra* and *Rxrb* deletion in mAMs and ImmAMs from 9-wk-old *Rxrab*^*fl/fl*^ and *Cd11c*^*Cre+*^*Rxrab*^*fl/fl*^ mice, *n* = 3 per genotype. **(F)** Percentages of CD45^+^ mAMs and ImmAMs in whole lung homogenates and BAL. *n* = 7–12 per genotype and method of isolation; data are from two independent experiments. **(G)** (Left) Representative flow cytometry dot plots showing the populations of DCs (cDC1 and cDC2), and (right) graphs showing the total number of cells per mg of tissue and the percentage of each DC population in 9-wk-old *Rxrab*^*fl/fl*^ and *LysM*^*Cre+*^*Rxrab*^*fl/fl*^ mice. *n* = 5–7 per genotype; data are from two independent experiments. **(H)** (Left) Representative flow cytometry dot plots showing IMs in 9-wk-old *Rxrab*^*fl/fl*^ and *LysM*^*Cre+*^*Rxrab*^*fl/fl*^, and (right) percentage of CD45^+^ cells. *n* = 3–7 per genotype; data are from two independent experiments. **(I)** (Left) Representative flow cytometry dot plots showing neutrophils (gated on Single/DAPI^−^/CD45^+^ cells) and eosinophils (gated on Single/DAPI^−^/CD45^+^/Cd11c^−^/Ly6G^−^ cells) in the whole digested lung, and (right) graph showing total numbers per mg of tissue from 9-wk-old *Rxrab*^*fl/fl*^ and *Cd11c*^*Cre+*^*Rxrab*^*fl/fl*^ mice. *n* = 7–8 per genotype and method of isolation; data are from two pooled independent experiments. **(J)** Quantification of PB neutrophils, eosinophils, and monocytes as (top) total number per μl blood and (bottom) percentage of CD45^+^ cells in 9-wk-old *Rxrab*^*fl/fl*^ and *Cd11c*^*Cre+*^*Rxrab*^*fl/fl*^ mice. *n* = 5–6 per genotype. **(K)** Lung Van Gieson histology images; scale bar, 250 μm. **(L)** (Left) FACS plots showing BODIPY^+^ mAMs, and (right) BODIPY mean fluorescence intensity (MFI) in mAMs from 9-wk-old *Rxrab*^*fl/fl*^ and *Cd11c*^*Cre+*^*Rxrab*^*fl/fl*^ mice. *n* = 8 per genotype; data are from two independent experiments. **(M)** Total protein (left) and phospholipid (right) content in BAL from 9-wk-old *Rxrab*^*fl/fl*^ and *Cd11c*^*Cre+*^*Rxrab*^*fl/fl*^ mice. *n* = 10-11 mice per genotype. **(N)** Protein-phospholipid ratio in BAL from 9-wk-old *Rxrab*^*fl/fl*^ and *Cd11c*^*Cre+*^*Rxrab*^*fl/fl*^ mice. *n* = 9–10 mice per genotype. All data are shown as means ± SEM; **P ≤ 0.01; ***P ≤ 0.001; ****P ≤ 0.0001; unpaired Student *t* test.

**Figure 1.**
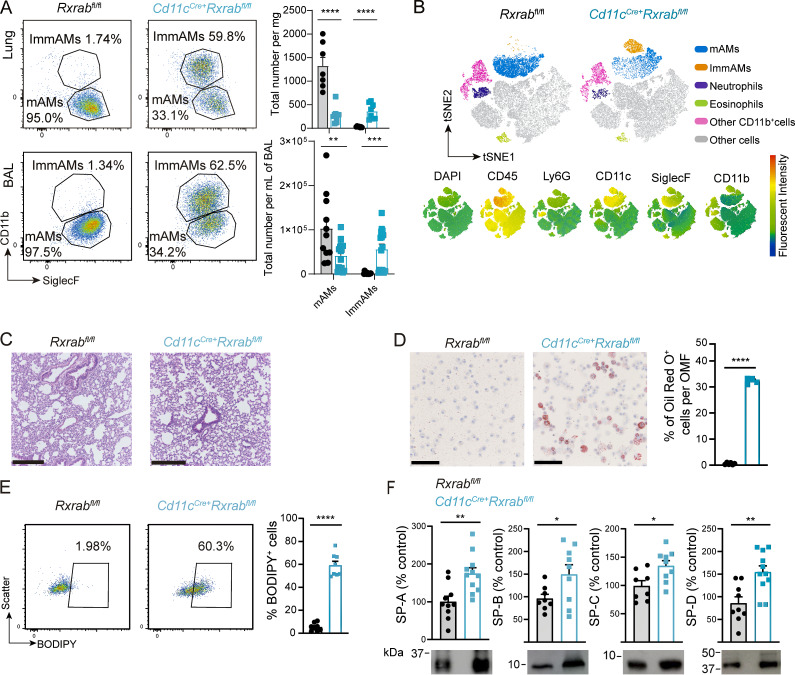
**Deletion of RXRα and RXRβ in AMs generates an immature cell phenotype and pulmonary proteinosis. (A)** (Left) Representative flow cytometry dot plots showing AMs gated onto CD11b and SiglecF in whole digested lung and BAL, and (right) graphs showing total numbers of mAMs and ImmAMs, per mg of tissue or ml of BAL, respectively. *n* = 7–12 per genotype and method of isolation; data from two independent experiments. **(B)** (Top) Annotated t-SNE plots generated with lung-homogenate FACS data for unbiased classification of the different cell populations found in CD45^+^ AMs (*n* = 3 per genotype) and (bottom) biexponential transformed selected marker expression levels. **(C)** Lung H&E histology images; scale bar, 250 μm. **(D)** (Left) Oil Red O staining of BAL cytospin cells; scale bar, 100 μm, and (right) percentage of Oil Red O^+^ cells per optical microscopy field (OMF). Quantifications were obtained by analysis of 10 OMFs per sample with *n* = 4–6 per genotype. **(E)** (Left) FACS plots showing BODIPY^+^ mAMs, and (right) quantification of BODIPY^+^ mAMs, with *n* = 8 per genotype; data representative from two independent experiments. **(F)** Quantification western blot of surfactant proteins A, B, C, and D, with *n* = 8–11 per genotype; data representative from two independent experiments. All data were obtained from 9-wk-old *Rxrab*^*fl/fl*^ and *Cd11c*^*Cre+*^*Rxrab*^*fl/fl*^ mice and are shown as means ± SEM; *P ≤ 0.05; **P ≤ 0.01; ***P ≤ 0.001; ****P ≤ 0.0001; unpaired Student *t* test. Source data are available for this figure: SourceData F1.

We first analyzed the expression of RXRα and RXRβ in circulating monocytes and AMs. Although AMs express both RXR subtypes, only *Rxra* showed increased expression compared with undifferentiated blood monocytes (Fig. S1 A). We previously reported that RXRα deletion with the pan-myeloid driver *LysM*^*Cre*+^ (*LysM*^*Cre*+^*Rxra*^fl/fl^ mice) produced no significant changes in AMs (Casanova-Acebes et al., 2020). However, several studies have shown that RXR redundancy allows one RXR subtype to compensate the lack of another (Rőszer et al., 2013; Casanova-Acebes et al., 2020; Menendez-Gutierrez et al., 2023). We therefore crossed *LysM*^*Cre*+^ mice with *Rxrab*^fl/fl^ mice to generate *LysM*^*Cre*+^*Rxrab*^fl/fl^ mice and analyzed lung homogenates by FACS, finding a reduction in the number of mature AMs (mAMs, CD11b^−^/SiglecF^+^) (Fig. S1, B–D). To generate a more specific model for the deletion of RXRs in AMs, we deleted *Rxra* and *Rxrb* using the *Cd11c*^*Cre*^ driver (Brocker et al., 1997), which provided efficient deletion in mAM and immature AM (ImmAM) populations (Fig. S1 E) without *Rxrg* upregulation (CT values >35, data not shown). The lack of RXRα and RXRβ signaling in AMs led to a pronounced reduction in the mAM population and a significant increase in ImmAMs (ImmAMs: CD11b^+^/SiglecF^+^) in both whole lung and bronchoalveolar lavage (BAL) of 9-wk-old mice (Fig. 1, A and B; and Fig. S1 F). The cDC1 and cDC2 populations and interstitial macrophages (IMs) remained unchanged under steady-state conditions in RXR-deficient lungs (Fig. S1, G and H). Analysis of other lung myeloid populations in *Cd11c*^*Cre+*^*Rxrab*^*fl/fl*^ mice revealed a reduction in the numbers of lung neutrophils, with no changes in eosinophils (Fig. 1 B and Fig. S1 I). BM hematopoiesis was maintained, and no changes were observed in peripheral blood (PB) neutrophil, eosinophil, or monocyte numbers (Fig. S1 J), suggesting that the reduction in lung neutrophils is due to impaired pulmonary recruitment of these cells in *Cd11c*^*Cre+*^*Rxrab*^*fl/fl*^ mice.

Histological analysis of lung sections demonstrated normal lung structure and alveolarization in *Cd11c*^*Cre+*^*Rxrab*^*fl/fl*^ mice (Fig. 1 C and Fig. S1 K). Notably, Oil Red O staining of BAL demonstrated a significant enrichment of foam cells (∼30% of total cells) (Fig. 1 D), and BODIPY staining showed significant lipid accumulation in RXR-deficient mAMs (Fig. 1 E and Fig. S1 L). Consistent with these findings, *Cd11c*^*Cre+*^*Rxrab*^*fl/fl*^ mice developed pulmonary alveolar proteinosis, evident from increased content of total proteins and phospholipids in BAL (Fig. S1 M), with no signs of lung edema (Fig. S1 N). Defective surfactant recycling by RXR-deficient AMs led to accumulation of all four surfactant proteins: SP-A, SP-B, SP-C, and SP-D (Fig. 1 F).

These results show that RXR signaling is required for the correct development of the AM population, including the reduction of CD11b surface marker expression. Additionally, the intracellular lipid accumulation in *Cd11c*^*Cre+*^*Rxrab*^*fl/fl*^ AMs demonstrates impaired AM function in the absence of RXRs, likely contributing to the accumulation of pulmonary alveolar surfactant proteins and phospholipids.

### RXR-deficient AMs have reduced survival linked to lipotoxicity

**Figure 2.**
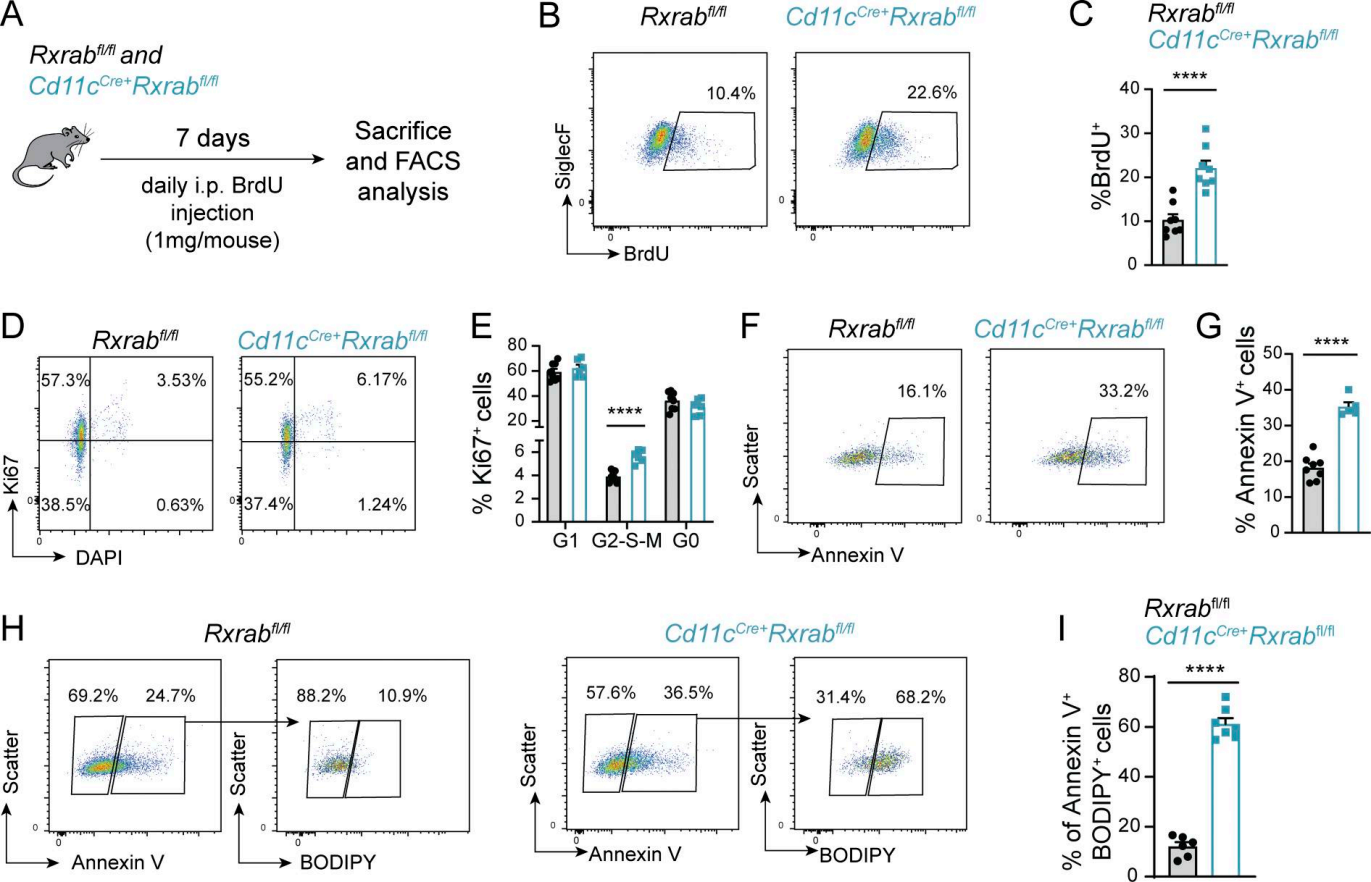
**RXR-depleted mAMs are more proliferative but have an exacerbated cell death rate linked to lipotoxicity. (A)** Scheme of BrdU assay. **(B)** Representative flow cytometry plots showing BrdU incorporation in mAMs. **(C)** Quantification of BrdU^+^ mAMs. *n* = 8 per genotype; data are from two pooled independent experiments. **(D)** Representative flow cytometry plots showing Ki67/DAPI staining in mAMs. **(E)** Quantification of cell cycle distribution analysis in mAMs according to Ki67 and DAPI levels. *n* = 6–8 per genotype; data are from two pooled independent experiments. **(F)** Representative flow cytometry plots showing Annexin V labeling in mAMs. **(G)** Quantification of Annexin V^+^ mAMs. *n* = 6–8 per genotype; data are from two pooled independent experiments. **(H)** Representative flow cytometry plots showing BODIPY stain analysis in Annexin V^+^ mAMs. **(I)** Quantification of Annexin V^+^/BODIPY^+^ mAMs. *n* = 6–7 per genotype; data are from two pooled independent experiments. All data were obtained from 9-wk-old *Rxrab*^*fl/fl*^ and *Cd11c*^*Cre+*^*Rxrab*^*fl/fl*^ mice and are shown as means ± SEM; analysis were performed based on FMO controls; ****P ≤ 0.0001; unpaired Student *t* test.

To dissect the mechanism by which AM numbers are reduced after RXR deletion, we studied the balance between proliferation and apoptosis. Daily BrdU administration for 7 days revealed a higher rate of proliferation by mAMs from *Cd11c*^*Cre+*^*Rxrab*^*fl/fl*^ mice (Fig. 2, A–C), and the increased proliferation capacity of RXR-deficient mAMs was confirmed by Ki-67 and DAPI staining. The mAM population in *Cd11c*^*Cre+*^*Rxrab*^*fl/fl*^ mice contained ∼2% more cells in G2-S-M phase than the equivalent population in *Rxrab*^*fl/fl*^ mice (Fig. 2, D and E), suggesting that RXR-deficient mAMs proliferate in an attempt to overcome the low cell numbers. Analysis of apoptosis by annexin V staining revealed a ∼15% higher proportion of apoptotic cells in the *Cd11c*^*Cre+*^*Rxrab*^*fl/fl*^ mAM population (Fig. 2, F and G). This result is in line with findings in RXR-deficient large cavity PMs (LPMs), which showed significant lipid accumulation in apoptotic cells (Casanova-Acebes et al., 2020). We found that the number of apoptotic cells with lipid accumulation was also higher in *Cd11c*^*Cre+*^*Rxrab*^*fl/fl*^ mAMs than in their *Rxrab*^*fl/fl*^ counterparts (Fig. 2, H and I). Within the *Cd11c*^*Cre+*^*Rxrab*^*fl/fl*^ mAM population, the number of lipid–laden apoptotic cells was almost double that of lipid-free apoptotic cells (Fig. 2 H). These results indicate that the lower AM numbers in RXR-deficient mice is the consequence of lipotoxicity-induced apoptosis.

### RXR controls postnatal AM maturation

**Figure 3.**
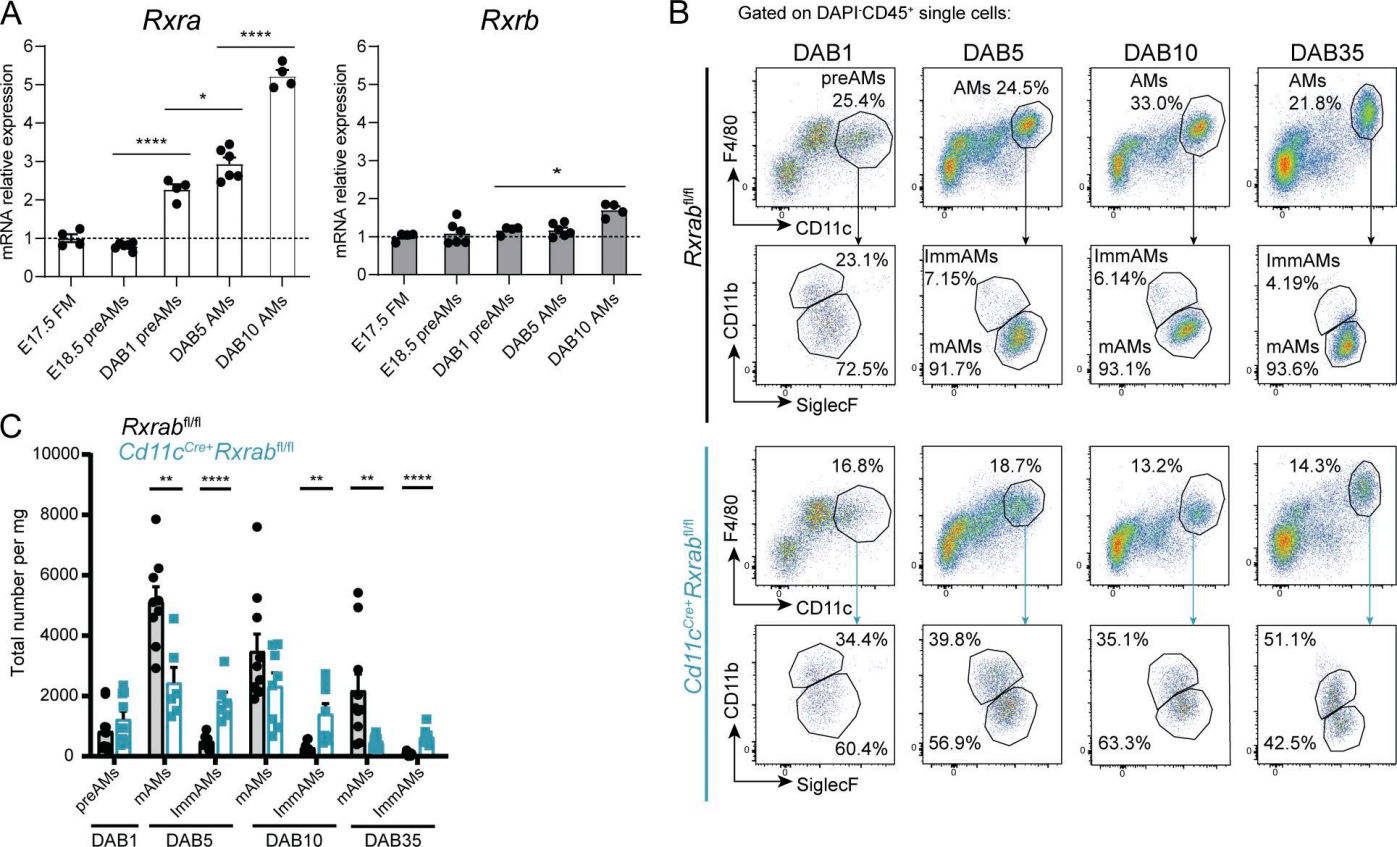
**RXRs are necessary for postnatal AM maturation. (A)** *Rxra* and *Rxrb* relative mRNA expression at different AM developmental stages, from embryonic fetal monocytes and preAMs to postnatal populations. *n* = 4–6 samples from four pooled mice; data are representative from two independent experiments. **(B)** Representative flow cytometry plots gated on alive CD45^+^ cells showing the expression of maturity markers in postnatal preAM and AM populations from *Rxrab*^*fl/fl*^ and *Cd11c*^*Cre+*^*Rxrab*^*fl/fl*^ mice. **(C)** Total numbers of the populations in B. *n* = 6–10 mice per genotype and stage; data are from two pooled independent experiments. All data are shown as means ± SEM; *P ≤ 0.05; **P ≤ 0.01; ****P ≤ 0.0001; (A) two-way ANOVA with Tukey’s multiple comparisons test; (C) unpaired Student *t* test.

Given the embryonic origin of AMs, we next explored the role of RXRs in early development. *Rxra* expression increased continuously from E18.5 to the first day after birth (DAB1) in preAMs and subsequently up to DAB10 in AMs (Fig. 3 A). The *Cd11c*^*Cre*^ model allows efficient recombination of floxed alleles from DAB5 (Schneider et al., 2014), allowing us to study the importance of RXR signaling for lung development in the postnatal period. Immediately after birth, AMs exist in a precursor state (preAMs), and Cre-mediated recombination of *Pparg* leads to the disruption of maturation into CD11b^−^/CD11c^+^ mAMs (Schneider et al., 2014). The DAB1 preAM populations segregated into two subpopulations according to CD11b maturity marker expression in both *Rxrab*^*fl/fl*^ and *Cd11c*^*Cre+*^*Rxrab*^*fl/fl*^ DAB1 mice (Fig. 3, B and C). However, by DAB5, *Rxrab*^*fl/f*^ preAMs had already downregulated CD11b surface marker expression and matured into AMs, whereas DAB5 *Cd11c*^*Cre+*^*Rxrab*^*fl/fl*^ preAMs failed to downregulate CD11b and did not fully acquire a mature phenotype (Fig. 3, B and C). This immature state due to impaired CD11b downregulation in *Cd11c*^*Cre+*^*Rxrab*^*fl/fl*^ mice was maintained through DAB35 (Fig. 3, B and C) and up to 9 wk (Fig. 1, A and B). Taken together, these results demonstrate the importance of RXRs for the correct transition from initial AM precursors to the fully mAM phenotype.

### RXRs regulate AM lipid metabolism, proliferation, and identity

**Figure 4.**
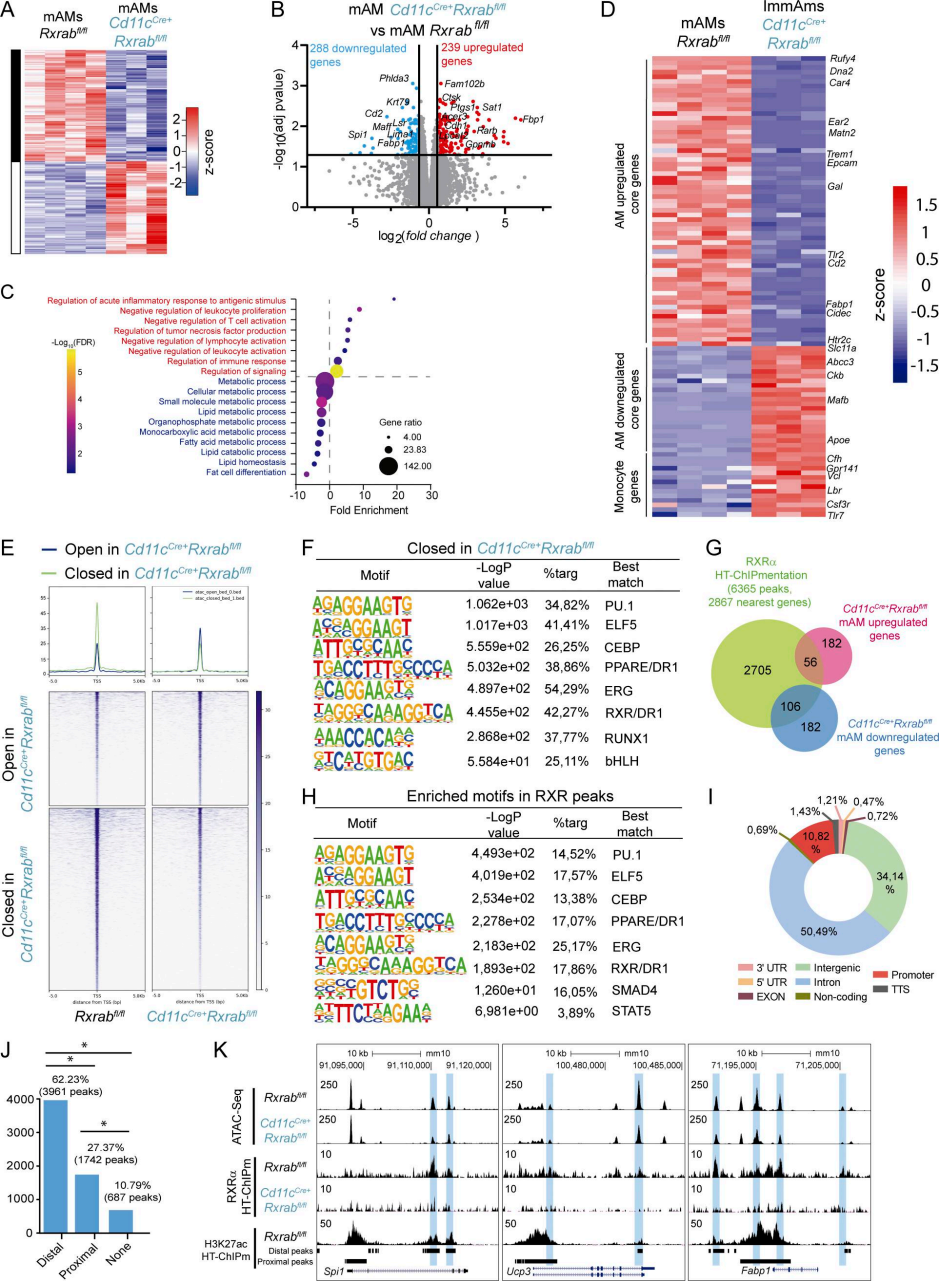
**RXRs act as LDTFs in AMs. (A)** Heatmap representing all DEGs detected in the comparison of *Rxrab*^*fl/fl*^ mAMs (*n* = 4 mice) with *Cd11c*^*Cre+*^*Rxrab*^*fl/fl*^ mAMs (*n* = 3 mice) from 9-wk-old mice. **(B)** Volcano plot showing global transcriptomic changes in *Cd11c*^*Cre+*^*Rxrab*^*fl/fl*^ mAMs. Colored dots represent DEGs with significant upregulation (p-adj <0.05 and FC >0; red) or significant downregulation (p-adj <0.05 and FC <0; blue). **(C)** Significantly enriched GO terms derived from the bulk RNA-seq data comparison between *Cd11c*^*Cre+*^*Rxrab*^*fl*/*fl*^ and *Rxrab*^*fl*/*fl*^ mAMs, detected with the PANTHER statistical enrichment test tool (P < 0.05 with Bonferroni correction). **(D)** Heatmap showing expression values of previously described typical AM-upregulated and -downregulated core genes and monocyte genes [20] in *Rxrab*^*fl/fl*^ mAMs (*n* = 4 mice) versus *Cd11c*^*Cre+*^*Rxrab*^*fl/fl*^ ImmAMs (*n* = 3 mice). **(E)** Genomic heatmap of ATAC-seq signals located in TSS-flanking regions (±5 kb) in sorted mAMs from 9-wk-old *Rxrab*^*fl*/*fl*^ and *Cd11c*^*Cre+*^*Rxrab*^*fl*/*fl*^ mice (*n* = 4 per genotype) (log2(FC) ≤ −1 or ≥1, FDR <0.05). **(F)** HOMER motif enrichment analysis in closed ATAC-seq peaks in *Cd11c*^*Cre+*^*Rxrab*^*fl*/*fl*^ mAMs. **(G)** Overlapping analysis for the collection of 2,867 genes that were identified as nearest to RXRα HT-ChIPmentation peaks and for the collection of 527 DEGs obtained in bulk RNA-seq. **(H)** HOMER motif enrichment analysis of RXR peaks. **(I)** Genome feature distribution analysis of RXR peaks. **(J)** Bar plot showing the association between RXRα peaks and H3K27ac peaks classified as proximal or distal peaks according to their distance from the nearest gene. **(K)** UCSC genome browser plots showing ATAC-seq, RXRα and H3K27ac (distal and proximal) HT-ChIPmentation peaks in key AM genes. Blue boxes label differentially accessible regions showing RXR binding in *Cd11c*^*Cre+*^*Rxrab*^*fl*/*fl*^ versus* Rxrab*^*fl*/*fl*^ mAMs. *P ≤ 0.05; Test of equal or given proportions (prop.test function in R).

**Figure 5.**
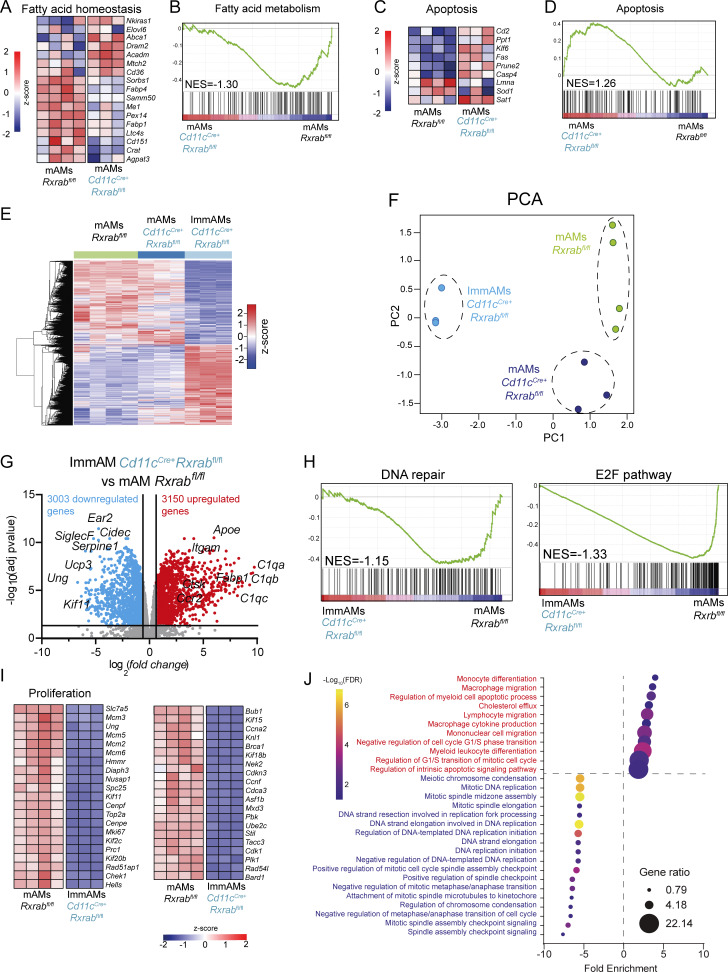
**Bulk RNA-seq analysis reveals that RXR signaling is necessary for AM identity, metabolism, and proliferation. (A)** Heatmap showing dysregulated genes related to fatty acid homeostasis in *Cd11c*^*Cre+*^*Rxrab*^*fl/fl*^ mAMs (*n* = 3 mice) versus *Rxrab*^*fl/fl*^ mAMs (*n* = 4 mice). **(B)** GSEA plot showing a negative normalized enrichment score (NES = −1.30) for the fatty acid metabolism gene set in *Cd11c*^*Cre+*^*Rxrab*^*fl/fl*^ mAMs versus *Rxrab*^*fl/fl*^ mAMs. **(C)** Heatmap showing dysregulated apoptosis-related genes in *Cd11c*^*Cre+*^*Rxrab*^*fl/fl*^ mAMs (*n* = 3 mice) versus *Rxrab*^*fl/fl*^ mAMs (*n* = 4 mice). **(D)** GSEA plot showing positive enrichment (NES = 1.26) for the apoptosis gene set. **(E)** Heatmap representing all DEGs obtained after the comparison of *Rxrab*^*fl/fl*^ mAMs (*n* = 4 mice) versus *Cd11c*^*Cre+*^*Rxrab*^*fl/fl*^ mAMs (*n* = 3 mice) versus *Cd11c*^*Cre+*^*Rxrab*^*fl/fl*^ ImmAMs (*n* = 3 mice) sorted from 9-wk-old mice. **(F)** PCA plot obtained using the transcriptomes of the previous samples. **(G)** Volcano plot showing global transcriptomic differences between *Rxrab*^*fl/fl*^ mAMs (*n* = 4 mice) and *Cd11c*^*Cre+*^*Rxrab*^*fl/fl*^ ImmAMs (*n* = 3 mice). Colored dots represent DEGs with significant upregulation (p-adj <0.05 and FC >0; red) or significant downregulation (p-adj <0.05 and FC <0; blue). **(H)** GSEA plots showing negative enrichment for the DNA repair and E2F pathway gene sets in *Cd11c*^*Cre+*^*Rxrab*^*fl/fl*^ ImmAMs versus *Rxrab*^*fl/fl*^ mAMs. **(I)** Heatmap showing proliferation-related dysregulated genes in the previous samples. **(J)** Significantly enriched GO terms derived from the bulk RNA-seq data comparison of *Cd11c*^*Cre+*^*Rxrab*^*fl*/*fl*^ ImmAMs versus *Rxrab*^*fl*/*fl*^ mAMs, detected with the PANTHER statistical enrichment test tool (P < 0.05 with Bonferroni correction).

We next investigated the molecular mechanism underlying the phenotype of RXR-deficient AMs by RNA sequencing (RNA-seq) in *Rxrab*^*fl/fl*^ mAMs and *Cd11c*^*Cre+*^*Rxrab*^*fl/fl*^ mAMs and ImmAMs. The analysis revealed differences between the three groups, which were further analyzed by pairwise comparisons (Table S1). RXR deficiency in Cd11c^*Cre+*^*Rxrab*^*fl*/*fl*^ mAMs was characterized by 527 differentially expressed genes (adjusted P value [p-adj] ≤0.05) (Fig. 4, A and B). Among the >280 downregulated genes were several AM core genes, including *Cd2*, *Cidec*, *Krt79*, *Cpne5,* and *Fabp1* (Gautier et al., 2012), as well as the myeloid master LDTF gene *Spi1* (Gautier et al., 2012) and other AM-specific TF-encoding genes such as *Lima1* and *Maff* (Gautier et al., 2012). The >230 upregulated genes included those encoding other metabolic enzymes such as *Fbp1* (Li et al., 2020) and *Sat1* (Fig. 4 B). PANTHER gene ontology (GO) term enrichment analysis indicated an association of the upregulated genes with cell activation and immune responses, whereas the downregulated genes were associated with metabolism-related terms, especially lipid metabolism (Fig. 4 C). Altered fatty-acid metabolism was confirmed by the specific detection of dysregulated genes (Fig. 5 A) and gene set enrichment analysis (GSEA) (Fig. 5 B). We also observed upregulation of genes involved in apoptosis (Fig. 5, C and D), confirming the results of the annexin V flow cytometry assays.

The largest transcriptional differences were found, however, in *Cd11c*^*Cre+*^*Rxrab*^*fl/fl*^ ImmAMs (Fig. 5, E and F), which had >6,000 dysregulated genes compared with *Rxrab*^*fl/fl*^ mAMs (Fig. 5 G). The >3,000 downregulated genes included AM core genes (*Ucp3* and *Cidec*) and AM canonical genes (*Siglecf*). Upregulated genes in *Cd11c*^*Cre+*^*Rxrab*^*fl/fl*^ ImmAMs included genes related to the complement response (*C1qa*, *C1qb*, and *C1qc*) and the monocyte gene *Ccr2* (Fig. 5 G). GSEA analysis revealed downregulation of DNA repair and the E2F proliferation pathway (Fig. 5 H), supported by the large number of downregulated proliferation-related genes (Fig. 5 I). GO term enrichment analysis with PANTHER revealed that upregulated genes were related to cell differentiation, proliferation, and division, whereas downregulated genes were related to DNA replication and the cell cycle (Fig. 5 J). The gene expression profile of *Cd11c*^*Cre+*^*Rxrab*^*fl/fl*^ ImmAMs was more similar to that of a monocyte than of a mAM, suggesting that these CD11b^+^ AMs correspond to a less differentiated state (Fig. 4 D). These results support a model in which RXRs are required for AM maturation, such that their loss leads to an undifferentiated ImmAM phenotype in which lipid metabolism and survival are compromised. In addition, RXR may act through heterodimeric transcriptional complexes to regulate these processes.

**Figure S2.**
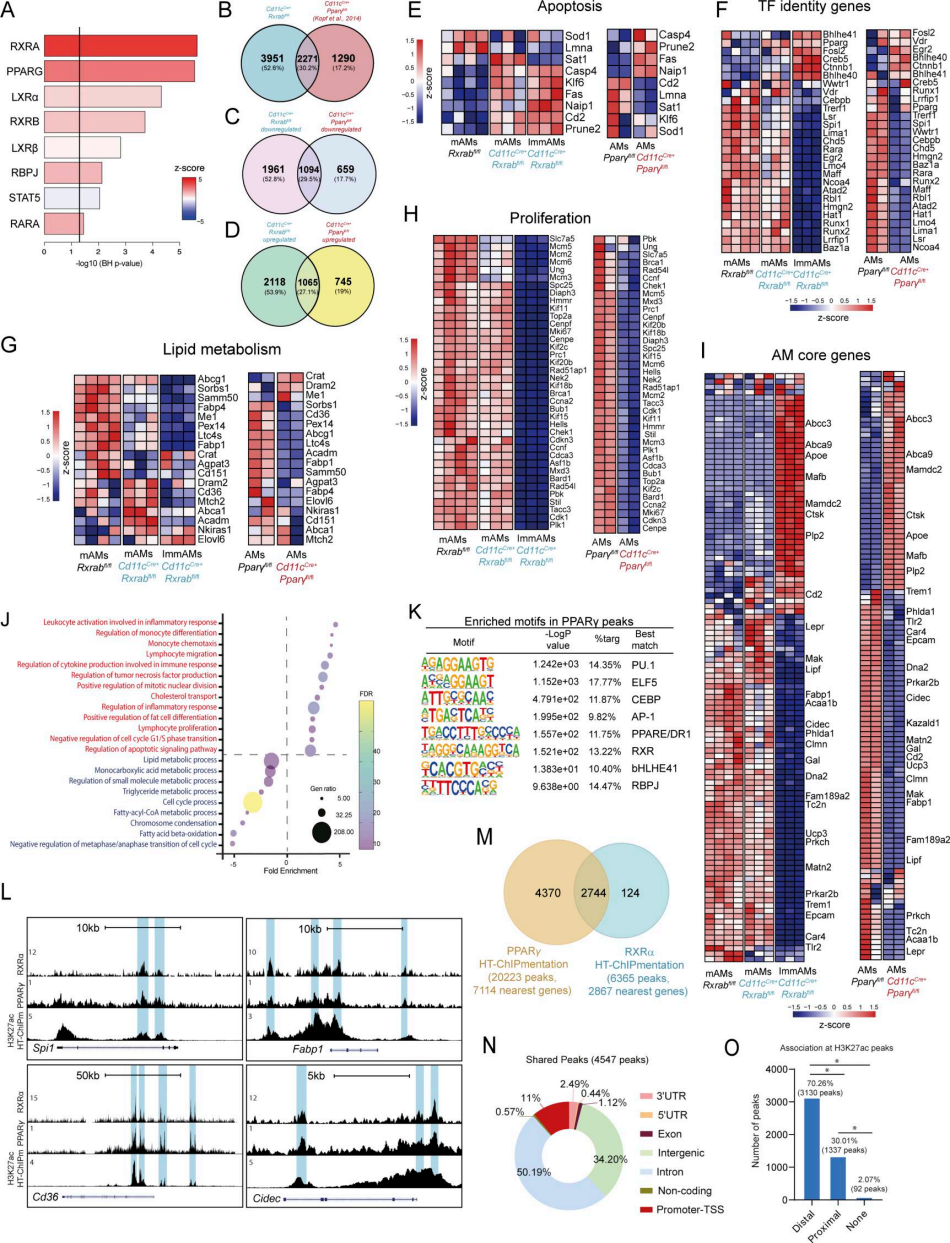
**Comparative transcriptomic analysis reveals RXR–PPARγ-driven co-regulation in AMs. (A)** Selected upstream transcriptional regulators identified by IPA of DEGs from the comparison between *Cd11c*^*Cre+*^*Rxrab*^*fl/fl*^ and *Rxrab*^*fl/fl*^ mAMs. Significant regulators were defined as –log10 (Benjamini–Hochberg P value) >1.3. **(B)** Venn diagram of overlaps between DEGs identified in *Cd11c*^*Cre+*^*Rxrab*^*fl/fl*^ mAMs (bulk RNA-seq) and DEGs from publicly available *Cd11c*^*Cre+*^*Pparγ*^*fl/fl*^ AM transcriptomic data. **(C and D)** Venn diagrams showing overlapping downregulated (C) and upregulated (D) genes between our *Cd11c*^*Cre+*^*Rxrab*^*fl/fl*^ RNA-seq dataset and the publicly available *Cd11c*^*Cre+*^*Pparγ*^*fl/fl*^ AM dataset. **(E–I)** Heatmap of dysregulated genes associated with selected biological processes in *Rxrab*^*fl/fl*^ mAMs, *Cd11c*^*Cre+*^*Rxrab*^*fl/fl*^ mAMs, and *Cd11c*^*Cre+*^*Rxrab*^*fl/fl*^ ImmAMs, compared with *Pparγ*^*fl/fl*^ AMs and *Cd11c*^*Cre+*^*Pparγ*^*fl/fl*^ AMs. **(J)** Significantly enriched GO terms derived from the comparison between *Cd11c*^*Cre+*^*Pparγ*^*fl*/*fl*^ and *Pparγ*^*fl*/*fl*^ AMs (PPARγ array data), identified using the PANTHER statistical enrichment test tool (P < 0.05, Bonferroni correction). **(K)** HOMER motif enrichment analysis of PPARγ peaks. **(L)** UCSC genome browser tracks showing RXRα, PPARγ, and H3K27ac HT-ChIPmentation peaks at representative AM genes. Blue boxes indicate regions with peaks identified across datasets. **(M)** Overlap analysis of genes nearest to PPARγ and RXRα peaks identified by HT-ChIPmentation. **(N)** Genomic feature distribution of the 4,547 overlapping PPARγ–RXRα peaks. **(O)** Bar plot showing the association between the 4,547 overlapping PPARγ–RXRα peaks and H3K27ac regions classified as proximal or distal according to distance from the nearest gene.*P <; Test of equal or given proportions (prop.test function in R).

Ingenuity pathway analysis (IPA) of upstream regulators identified RXRs and PPARγ as the most significant transcriptional regulators, supporting the idea that the RXR–PPARγ heterodimer controls many AM core genes, as well as those involved in AM-specific TF-encoding programs, lipid metabolism, apoptosis, and proliferation (Fig. S2 A). To further investigate co-regulation of these genes in AMs, we performed a comparative transcriptomic analysis integrating our RNA-seq data with publicly available PPARγ expression data in AMs (GSE60249) (Fig. S2, B–D). The gene expression profile confirmed co-regulation of numerous genes (Fig. S2, E–I). PANTHER GO enrichment analysis revealed functional similarities with our bulk RNA-seq data. Upregulated genes were predominantly linked to cell differentiation, migration, proliferation, apoptosis, and division, while downregulated genes were associated with cell cycle regulation and lipid metabolism (Fig. S2 J). These findings suggest that RXR–PPARγ heterodimerization plays a central role in orchestrating key biological processes in AMs.

**Figure S3.**
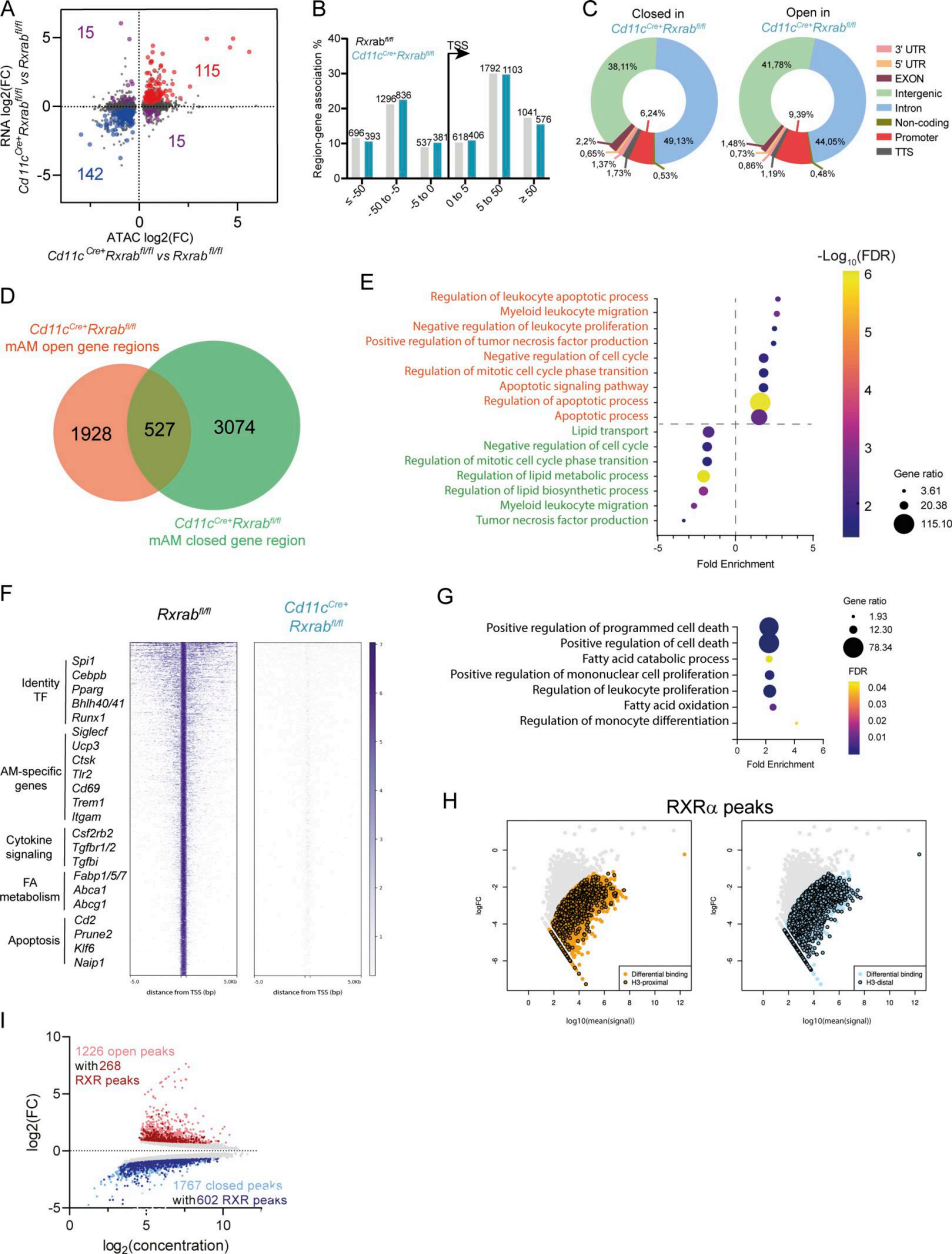
**ATAC-seq and genome-wide binding show the importance of RXR signaling for AM function, identity, and chromatin regulation. (A)** Dot plot showing the correlation between the logFC values of RNA-seq–based DEGs and the logFC values of differentially accessible regions (DARs) obtained by ATAC-seq in *Cd11c*^*Cre+*^*Rxrab*^*fl*/*fl*^ ImmAMs versus *Rxrab*^*fl*/*fl*^ mAMs. DARs and DEGs were paired according to the nearest gene detected for each DAR. **(B)** Distribution of nearest gene TSS distances for DARs in *Cd11c*^*Cre+*^*Rxrab*^*fl*/*fl*^ ImmAMs and *Rxrab*^*fl*/*fl*^ mAMs. **(C)** Distribution of genomic feature associations for DARs in *Cd11c*^*Cre+*^*Rxrab*^*fl*/*fl*^ ImmAMs and *Rxrab*^*fl*/*fl*^ mAMs. **(D)** Venn diagram comparing the collections of genes identified as nearest to open and closed DARs, as identified by ATAC-seq. **(E)** Significantly enriched GO terms detected with the PANTHER statistical enrichment test tool (P < 0.05 with Bonferroni correction) for the collections of genes exclusively associated with closed regions (green) or open regions (orange), as described by the Venn diagram in E. **(F)** Genomic heatmaps of RXRα binding signals located in TSS-flanking regions (±5 kb) in sorted mAMs from 9-wk-old *Rxrab*^*fl*/*fl*^ and *Cd11c*^*Cre+*^*Rxrab*^*fl*/*fl*^ mice (*n* = 2 per genotype) (FC ≤ −2 or ≥2, FDR <0.05). **(G)** Significantly enriched GO terms from RXR-controlled genes detected with the PANTHER statistical overrepresentation test tool (P ≤ 0.05 with Bonferroni correction). **(H)** MA plots for RXRα ChIPmentation peaks. Orange and blue dots represent 6,365 peaks immunoprecipitated with anti-RXRα antibody exclusively in *Rxrab*^*fl*/*fl*^ mAMs. Dots outlined in black represent peaks that overlap with H3K27ac regions classified as proximal to genes (left plot) or distal (right plot). **(I)** MA plot showing the overlap between differentially accessible ATAC-seq peaks (FDR <0.05) with RXRα HT-ChIPmentation peaks in mAMs. Colored dots correspond to differentially accessible ATAC-seq peaks with a Log2(FC)xLog2(concentration) value ≥0.65; light red, differentially open peaks with no RXR peak; light blue, differentially closed peaks with no RXR peak; dark red, differentially open peaks colocalizing with RXR peaks; dark blue, differentially closed peaks colocalizing with RXR peaks.

To investigate the regulatory landscape of RXRs in AMs, we assayed chromatin accessibility by assay for transposase-accessible chromatin sequencing (ATAC-seq), revealing a marked correlation between chromatin accessibility and RNA-seq–based gene expression profiles (Fig. S3 A). Of the 9,662 differentially accessible regions identified, 5,962 were less accessible in the RXR-depleted mAMs (false discovery rate [FDR] ≤ 0.05), demonstrating a more closed chromatin status (Fig. 4 E and Table S2). Analysis of the distribution of distances between differentially accessible regions and the nearest gene transcription start site (TSS) revealed no significant differences between *Rxrab*^*fl/fl*^ and *Cd11c*^*Cre+*^*Rxrab*^*fl/fl*^ mAMs, and similarly there were no differences in the association of these regions with a variety of genomic features (Fig. S3, B and C). To examine the functions potentially affected by the redistribution of chromatin accessibility in RXR-depleted mAMs, we compared the lists of genes associated, by proximity, with differentially accessible regions, defining those genes that were exclusively associated with open regions (1,928 genes) or closed regions (3,074 genes) in RXR-deficient mAMs (Fig. S3 D). Consistent with the RNA-seq analysis and phenotypic characterization, functional enrichment analysis revealed that genes exclusively linked to open regions were associated with terms related to apoptosis, migration, or proliferation, whereas genes exclusively linked to closed regions were associated with terms related to lipid metabolism (Fig. S3 E). Motif enrichment analysis showed that closed regions in *Cd11c*^*Cre+*^*Rxrab*^*fl*/*fl*^ mAMs were enriched in binding sites for key TFs for AM identity, including PU.1, ELF5, CEBP, PPAR, RXR, bHLH, and RUNX1 (McCowan et al., 2021; Sajti et al., 2020) (Fig. 4 F).

These alterations provide evidence that RXRs are key regulators of AM chromatin landscape and identity. To confirm this, we generated a genome-wide profile of RXR binding in mAMs by high-throughput ChIP combined with tagmentation-based library preparation (HT-ChIPmentation) (Gustafsson et al., 2019) in *Rxrab*^*fl*/*fl*^ and *Cd11c*^*Cre+*^*Rxrab*^*fl*/*fl*^ mAMs (Table S3). Immunoprecipitation with anti-RXRα antibody identified 6,365 regions exclusive to *Rxrab*^*fl*/*fl*^ mAMs, and a collection of 2,867 genes was identified as nearest to RXR-bound regions. Comparison of this gene collection with the genes detected as differentially expressed in bulk RNA-seq of *Cd11c*^*Cre+*^*Rxrab*^*fl*/*fl*^ mAMs suggested that RXRs directly control ∼30% of the dysregulated genes (Fig. 4 G). Among the genes assumed to be directly controlled by RXRs, we identified key TFs for AM specification (*Spi1*, *Cebpb*, *Pparg*, *Bhlhe40*, *Bhlhe41*, and *Runx1*), AM identity genes (*Siglecf*, *Ucp3*, *Ctsk*, *Cd69*, and *Tlr2*), receptors for AM-shaping cytokines (*Csf2r2b* and *Tgfbr1*/*2*), lipid metabolism genes (*Fabp1*, *Abca1*, and *Abcg1*), and apoptosis-related genes (*Cd2*, *Prune*, *Klf6*, and *Naip1*) (Fig. S3 F). Consistent with the phenotypic characterization, the genes directly controlled by RXRs were associated with terms related to cell death, proliferation, differentiation, and lipid metabolism (Fig. S3 G). Motif enrichment analysis for RXR-bound regions revealed RXR colocalization with many of the key TFs for AM identity, including STAT5 and SMAD4, the TFs downstream of GM-CSF and TGFβ (Fig. 4 H). This finding supports the idea that cooperation of RXRs with other TFs is needed to maintain the mAM regulatory landscape and mAM identity. Genome distribution analysis revealed that 10.82% of RXR peaks were located in promoters, 34.4% in intergenic regions, and 50% in intronic regions, indicating RXR action through enhancers in mAMs (Fig. 4 I). In parallel, we ran a histone H3 lysine 27 acetylation (H3K27ac) HT-ChIPmentation assay in mAMs, classifying H3K27ac peaks located outside a ±3 kb window around a TSS as putative enhancers (Table S4). The intersection of RXRα peaks with distance-discriminated H3K27ac peaks showed that RXR-binding sites were significantly more frequently associated with H3K27ac distal sites, implying that the most frequent interactions of RXRs are through putative enhancers (Fig. 4 J and Fig. S3 H). To gain comprehensive insight into the importance of RXR interactions and chromatin accessibility programming, we overlapped the differentially accessible ATAC-seq and RXRα HT-ChIPmentation peaks, revealing that ∼22% of the open peaks in *Cd11c*^*Cre+*^*Rxrab*^*fl*/*fl*^ mAMs coincided with RXR peaks. The coincidence increased to ∼34% for the closed peaks in *Cd11c*^*Cre+*^*Rxrab*^*fl*/*fl*^ mAMs (Fig. S3 I). ATAC-seq differential accessibility correlated with RXR and H3K27ac peaks for key identity TFs, AM-specific genes, and metabolic genes, exemplified, respectively, by *Spi1*, *Ucp3*, and *Fabp1* (Fig. 4 K). To address whether the regulatory network is associated with PPARγ, we performed PPARγ HT-ChIPmentation (Table S5), finding that PPARγ-binding overlapped with RXR occupancy, consistent with RXR–PPARγ heterodimer-mediated transcriptional regulation (Fig. S2, K–O). These results substantiate the role of RXRs as key TFs in AMs, essential for the maintenance of a precise chromatin regulatory landscape and for the expression of the receptors for instructive cytokines and differentiation TFs.

### RXR signaling is required for AM differentiation and maturation

**Figure 6.**
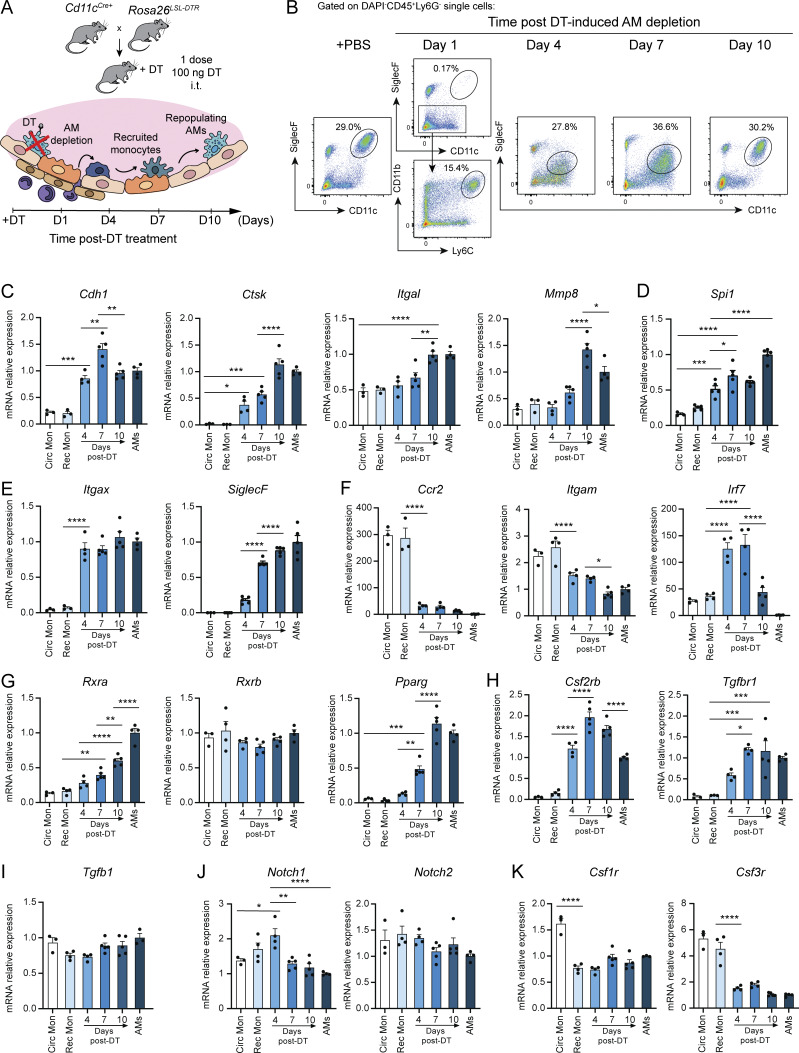
**Differentiating recruited monocytes increased expression of AM-specific genes and *Rxra* upon AM depletion. (A)** DT treatment for AM depletion in *Cd11c*^*Cre+*^*R26*^*LSL-DTR*^ mice. **(B)** Representative flow cytometry plots illustrating AM repopulation from recruited monocytes after AM depletion. **(C–K)** Gene expression patterns in circulating monocytes (Circ Mon), recruited monocytes (Rec Mon), repopulating/differentiating cells at 4–10 days after DT, and mAMs, *n* = 3–5 mice per group. Data are representative of two independent experiments and presented as mean ± SEM; *P ≤ 0.05; **P ≤ 0.01; ***P ≤ 0.001; ****P ≤ 0.0001; one-way ANOVA.

Having shown the critical role of RXRs in AM maturation and identity, we next investigated if RXR signaling was also necessary for differentiation. For this, we characterized the recruitment of circulating monocytes into the lung and their differentiation into mAMs by crossing *Cd11c*^*Cre+*^ mice with R26^LSL-DTR^ mice. Following intratracheal administration of diphtheria toxin (DT), we analyzed myeloid populations in the lungs of Cd11c^Cre+^R26^LSL-DTR^ mice at 1, 4, 7, and 10 days (Fig. 6 A). DT treatment resulted in specific depletion of AMs and the recruitment of circulating monocytes within the first 24 h (Fig. 6 B). Repopulating AM-like cells continued to increase their CD11c and SiglecF expression at days 4 and 7 after DT. By 10 days after DT administration, repopulating cells differentiated into indistinguishable AMs (Fig. 6 B). We next measured key gene expression kinetics in monocytes, recruited monocytes, differentiating cells, and AMs. The recruited monocytes and repopulating AMs progressively acquired a gene expression profile similar to that of mature steady-state AMs. Differentiation and maturation over 10 days after DT treatment were accompanied by increasing expression of AM-core genes, including *Cdh1*, *Ctsk*, *CD11a*, and *Mmp8* (Fig. 6 C); the pan-macrophage identity TF PU.1, encoding gene *Spi1* (Fig. 6 D); and other AM-specific genes such as *Cd11c* and *SiglecF* (Fig. 6 E). Conversely, the expression of the monocyte-specific *Ccr2* and *CD11b* were downregulated in the repopulating AMs (Fig. 6 F). Irf7, whose expression is needed for AM differentiation and development (Ning et al., 2011), was transiently upregulated in the first days after DT administration, but its expression had decreased by day 10 after DT (Fig. 6 F). Rxra expression also increased during the differentiation of recruited monocytes into AMs, suggesting that RXRα is needed for AM maturation and differentiation, whereas Rxrb expression was unaltered (Fig. 6 G). The expression of the RXR partner PPARγ (*Pparg*) increased (Fig. 6 G). The expression of the RXR targets *Csf2rb* and *Tgfbr1*, encoding the GM-CSF and TGFβ receptors, was increased upon recruitment of monocytes to the lung (Fig. 6 H). *Tgfb1* gene expression was unaltered during differentiation (Fig. 6 I); however, *Notch1*, but not *Notch2*, was transiently upregulated (Fig. 6 J), suggesting a role of the Notch pathway during the initial differentiation stages. In addition, the M-CSF and G-CSF receptor genes, *Csf1r* and *Csf3r*, showed significant declines in expression following cell differentiation (Fig. 6 K). These results support the idea that lung-recruited monocytes gain a distinct gene expression pattern that includes AM-specific genes, cytokine receptors, and *Rxra* expression during their differentiation into mAMs.

**Figure 7.**
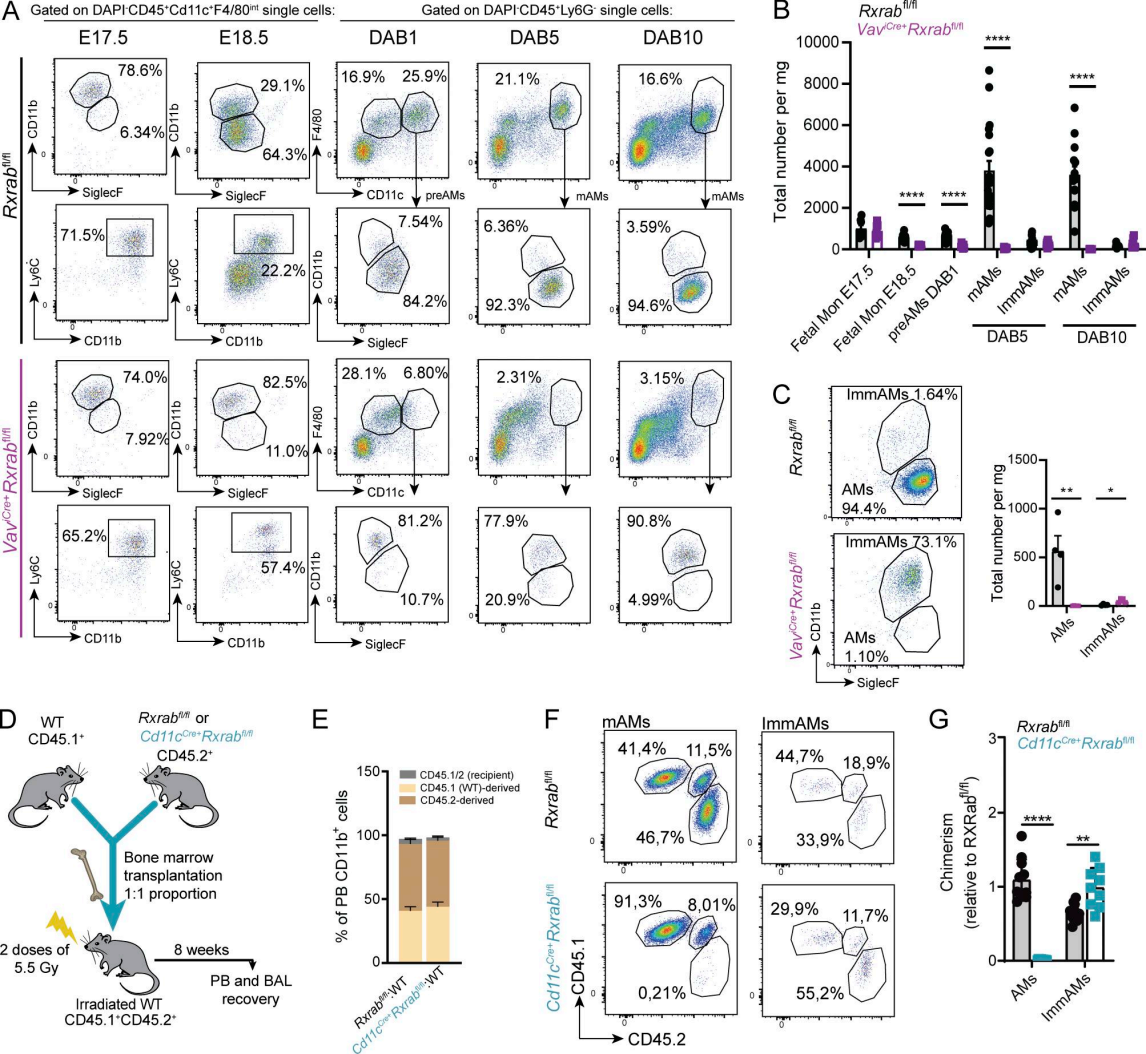
**RXR signaling is necessary for correct AM differentiation and maturation. (A)** Representative flow cytometry plots showing embryonic and postnatal preAM and AM populations. **(B)** Quantification of A, showing total cell numbers of the selected populations in *Rxrab*^*fl/fl*^ and *Vav*^*iCre+*^*Rxrab*^*fl/fl*^ mice. *n* = 7–10 per genotype from two independent experiments. **(C)** (Left) Representative flow cytometry plots showing AM subpopulations and (right) quantification in 9-wk-old *Rxrab*^*fl/fl*^ and *Vav*^*iCre+*^*Rxrab*^*fl/fl*^ mice. *n* = 4–5 per genotype; data representative from two independent experiments. **(D)** Competitive BM transplant procedure. **(E)** Quantification of PB chimerism in CD11b^+^ cells. **(F)** Representative flow cytometry plots showing mAMs and ImmAMs in lungs after BM transplant. **(G)** Quantification of *Rxrab*^*fl/fl*^ versus *Cd11c*^*Cre+*^*Rxrab*^*fl/fl*^-derived mAMs and ImmAMs after BM transplant. *n* = 9–10 per genotype; data are from two pooled independent experiments. All data are shown as means ± SEM; *P ≤ 0.05; **P ≤ 0.01; ****P ≤ 0.0001; unpaired Student *T* test.

The pattern of increasing *Rxra* expression in preAMs/AMs after birth (Fig. 3 A) and in differentiating recruited cells (Fig. 6 G) suggests that RXR signaling is necessary for AM differentiation and maturation. To confirm this, we used two approaches. First, we deleted RXRs during embryogenesis to study AM differentiation; for this, we used the *Vav*^*iCre*+^ model, which drives efficient recombination in all hematopoietic cells (Georgiades et al., 2002). Early deletion of *Rxra* and *Rxrb* led to impaired differentiation and maturation of preAMs from E18.5 (Fig. 7, A and B). During postnatal development, the few remaining AMs were found exclusively with an immature phenotype up to 9 wk (Fig. 7, B and C). These results indicate that RXR signaling is necessary for embryonic and postnatal AM maturation and differentiation. Second, BM from either *Rxrab*^*fl/fl*^ or *Cd11c*^*Cre+*^*Rxrab*^*fl/fl*^ CD45.2^+^ mice was combined with BM from WT CD45.1^+^ competitor mice in a 1:1 proportion and transplanted into lethally irradiated CD45.1^+^ CD45.2^+^ recipient mice (Fig. 7 D). No differences were observed in PB chimerism between recipients of *Rxrab*^*fl/fl*^ or *Cd11c*^*Cre+*^*Rxrab*^*fl/fl*^ BM (Fig. 7 E). Transplanted *Cd11c*^*Cre+*^*Rxrab*^*fl/fl*^ BM cells failed to reconstitute the mAM population when in competition with WT cells (Fig. 7, F and G). Moreover, the ImmAM population was enriched in recipients of *Cd11c*^*Cre+*^*Rxrab*^*fl/fl*^-derived cells (Fig. 7, F and G). This result shows that the deletion of RXR confers a competitive disadvantage for AM maturation. Together, our findings strongly support the idea that RXR signaling is critical for AM maturation and differentiation from embryonic development, through neonatal stages, and into adult life.

### GM-CSF, TGFβ, and DLL4 induce RXR expression in AMs

**Figure S4.**
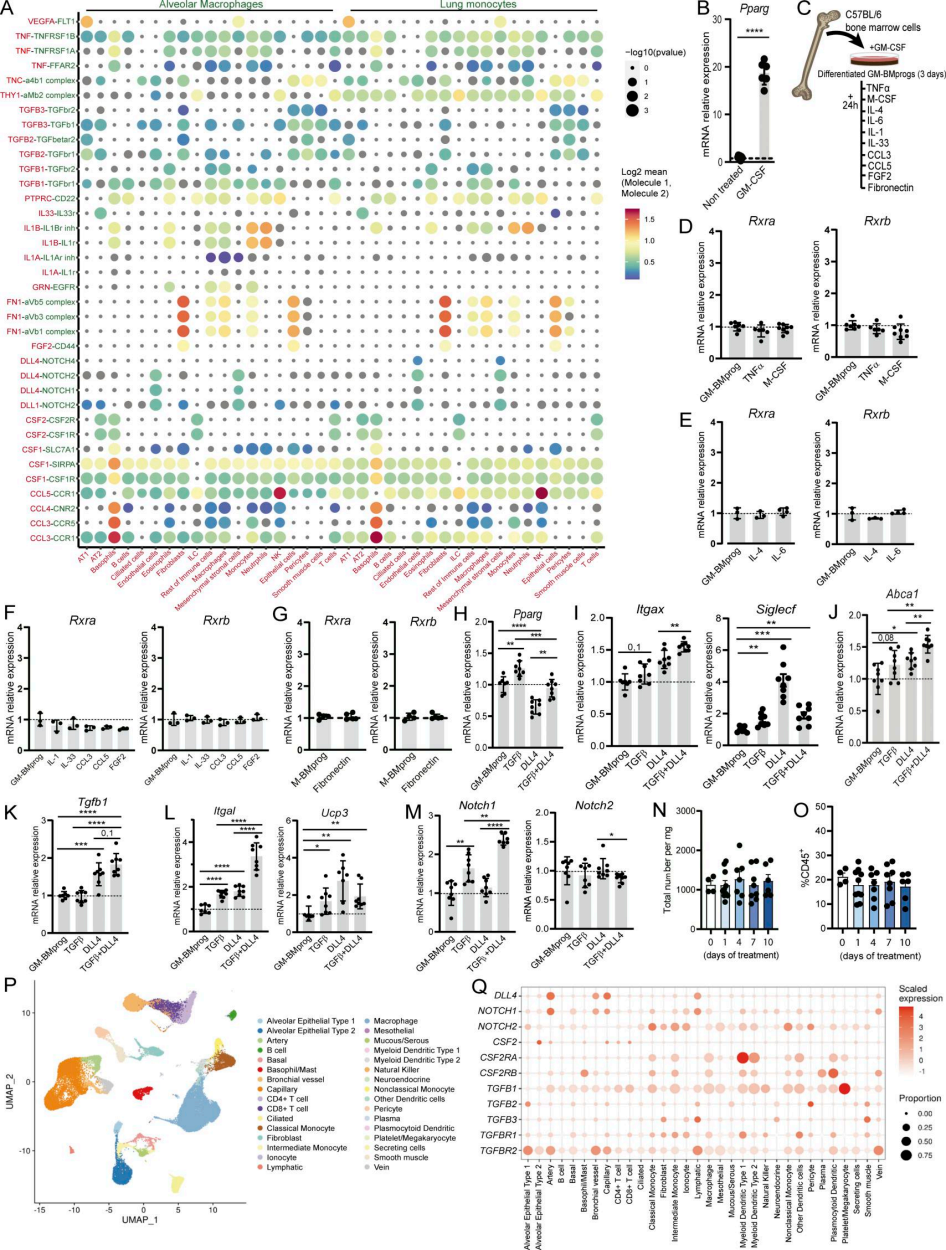
**Cell-to-cell communication analysis reveals DLL4 signaling as a key niche signal for Rxr gene induction and AM maturation. (A)** Bubble plot showing CellPhoneDB-identified ligand–receptor interaction pairs for different lung cell pairs. Ligands and secretor/surface-expressing cells are in red type, receptors and receptor cells in green type. The color code represents the Log2 mean expression value of the cytokine/receptor genes in the predicted interacting cells. **(B)** Normalized mRNA expression of *Pparg* in BM progenitors treated with GM-CSF for 72 h. *n* = 5–6 per condition. **(C)** Experimental design of the treatment with different cytokines of GM-CSF differentiated BM progenitors (GM-BMprog). **(D–G)** Normalized mRNA expression of *Rxra* and *Rxrb* in differentiated GM-BMprogs treated with the indicated cytokines for 24 h. *n* = 3–8 per condition; data are from up to two independent experiments. **(H–M)** Normalized mRNA expression of different genes in GM-BMprogs treated with TGFβ, DLL4, or TGFβ+DLL4 for 24 h. *n* > 7 per condition; data are from two pooled independent experiments. **(N and O)** Quantification of AMs as total cell number (N) and the percentage of CD45^+^ cells (O) after LY411575 treatment for 0, 1, 4, 7, or 10 days. *n* > 4 per condition; data are from two independent experiments. **(P)** UMAP plots showing the analyzed cell populations identified in publicly available scRNA-seq data of human lung. **(Q)** CellPhoneDB plot analyzing the expression of different signals and receptors in human lung cells. All data are shown as means ± SEM; *P ≤ 0.05; **P ≤ 0.01; ***P ≤ 0.001; ****P ≤ 0.0001; (B–M) unpaired Student *t* test; (N and O) one-way ANOVA with Tukey’s multiple comparisons test.

**Figure 8.**
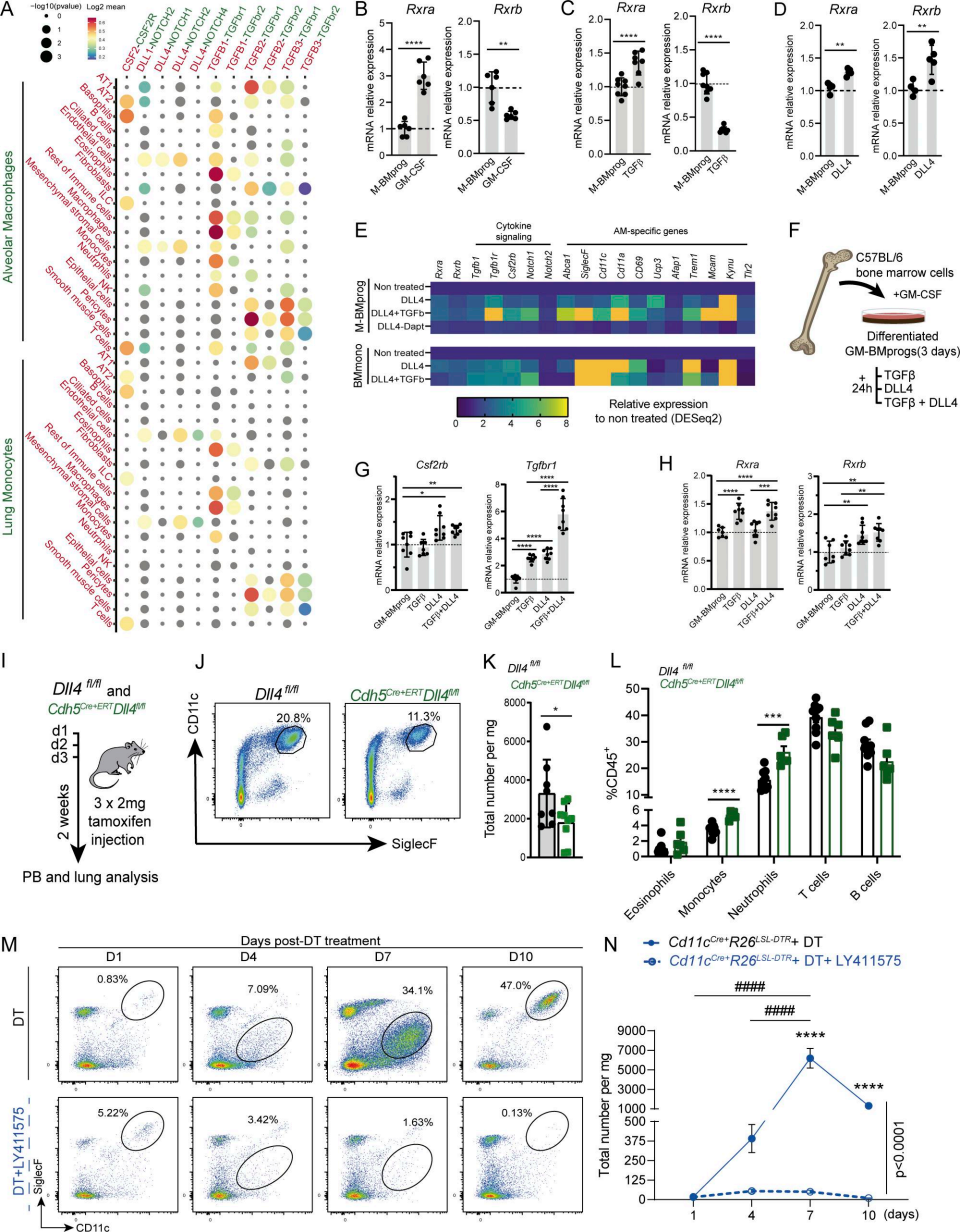
**GM-CSF, TGFβ, and DLL4 are necessary for AM differentiation and maturation. (A)** Bubble plot showing CellPhoneDB-identified ligand–receptor interaction pairs for different lung–cell pairs. Ligands and secretor/presenter cells are in red type, receptors and receptor cells in green type. The color code represents the Log2 mean expression values of the cytokine and receptor genes in the predicted interacting cells. **(B)** Normalized mRNA expression of *Rxra* and *Rxrb* in M-CSF–derived BM progenitors (M-BMprog) treated with GM-CSF for 72 h. *n* = 5–6 per condition; data are representative from two independent experiments. **(C)** Normalized mRNA expression of *Rxra* and *Rxrb* in M-BMprogs treated with TGFβ for 24 h. *n* = 7–8 per condition; data are representative from two independent experiments. **(D)** Normalized mRNA expression of *Rxra* and *Rxrb* in M-BMprogs treated with DLL4 for 24 h. *n* = 4–5 per condition; data are representative from two independent experiments. **(E)** Heatmap showing AM-related genes in M-BMprogs and BM monocytes (BMmono) treated with DLL4 and/or TGFβ or DLL4-Dapt. Orange values are >8. **(F)** Experimental design for the treatment of GM-CSF–derived BM progenitors (GM-BMprogs) with TGFβ, DLL4, or both molecules for 24 h. **(G)** Normalized mRNA expression of AM cytokine receptors. *n* > 7 per condition; data are from two pooled independent experiments. **(H)** Normalized mRNA expression of *Rxra* and *Rxrb. n* > 7 per condition; data are from two pooled independent experiments. **(I)** Experimental design of tamoxifen injection in *Dll4*^*fl/fl*^ and *Cdh5*^*Cre+ERT*^*Dll4*^*fl/fl*^ mice. **(J)** Representative flow cytometry plot of AMs gated on Single/DAPI^−^/CD45^+^/Ly6G^−^ cells in tamoxifen-treated *Dll4*^*fl/fl*^ and *Cdh5*^*Cre+ERT*^*Dll4*^*fl/fl*^ adult mice. **(K)** AM quantification in 11-wk-old *Dll4*^*fl/fl*^ versus *Cdh5*^*Cre+ERT*^*Dll4*^*fl/fl*^ mice. **(L)** PB leukocyte populations as a percentage of CD45^+^ cells in tamoxifen-treated *Dll4*^*fl/fl*^ and *Cdh5*^*Cre+ERT*^*Dll4*^*fl/fl*^ adult mice. *n* > 6 per genotype; data are from two pooled independent experiments. **(M)** Representative flow cytometry dot plots of lung homogenates (based on Fig. 8 J gating strategy), showing AM recovery after treatment with DT or DT+LY411575. **(N)** Total numbers of AMs after DT or DT+LY411575 treatment. *n* = 5–8 per genotype; data are from two independent experiments. All data are shown as means ± SEM; *P ≤ 0.05; **P ≤ 0.01; ***P ≤ 0.001; ****P ≤ 0.0001; ####P ≤ 0.0001; (B–D and K–L) unpaired Student *t* test; (G and H) one-way ANOVA with Tukey’s multiple comparisons test; (N) two-way ANOVA with Tukey’s multiple comparisons test.

To investigate the niche signals responsible for RXR gene induction in AMs, we analyzed cell-to-cell communication in the lung niche through a CellPhoneDB-based analysis (Efremova et al., 2020) using publicly available single-cell RNA-seq (scRNA-seq) data (Cohen et al., 2018). This analysis identified significant putative ligand–receptor pairs signaling either to AMs or to lung monocytes (Fig. S4 A). These interactions indicated that the known instructive niche-derived signals GM-CSF and TGFβ were signaling to AMs (Fig. 8 A), corroborating previous reports (Gschwend et al., 2021; Schneider et al., 2014; Cohen et al., 2018; Yu et al., 2017). We next differentiated BM progenitors by treating them for 3 days with M-CSF (generating M-BMprogs), followed by treatment with GM-CSF or TGFβ. Both treatments induced *Rxra* (Fig. 8, B and C) but failed to induce *Rxrb*, likely because *Rxrb* expression is characteristic of less committed cells (Menendez-Gutierrez et al., 2023). The treatment with GM-CSF also induced the *Pparg* gene (Fig. S4 B). Other putative interactions predicted by CellPhoneDB included TNFα, M-CSF, IL-4, IL-6, IL-1, IL-33, CCL3, CCL5, FGF2, and fibronectin (Fig. S4 A). None of these signals induced *Rxra* or *Rxrb* in differentiating progenitors (Fig. S4, C–G). Another ligand–receptor pairing identified in the CellPhoneDB analysis was endothelial DLL4 signaling to lung monocytes through NOTCH receptors (Fig. 8 A). DLL4 signaling has been shown to induce the expression of key LDTFs in differentiating monocytes and to be indispensable for Kupffer cell identity (Sakai et al., 2019; Bonnardel et al., 2019). Using RT-qPCR, we confirmed that *in vitro* DLL4 signaling induced *Rxra and Rxrb* expression in M-BMprogs (Fig. 8 D). Analysis of published RNA-seq data on M-BMprogs and BM monocytes demonstrated that cooperation between DLL4 and TGFβ can induce *Rxra* and *Rxrb* expression (Sakai et al., 2019) (Fig. 8 E). These cytokines also induced the RXR target genes *Csf2rb* and *Tgfb1r*, as well as other AM-related genes such as *Siglecf*, *Cd11c*, *Cd11a*, *Mcam*, and *Kynu* (Gautier et al., 2012) (Fig. 8 E). Considering the prevalence of GM-CSF expression in the alveolar niche, we also conducted *in vitro* experiments using GM-CSF as the background signal for progenitor differentiation. We differentiated BM progenitors with GM-CSF (GM-BMprogs) for 3 days and then tested the effect of exposure to TGFβ, DLL4, or both signals simultaneously (Fig. 8 F). Treatment with DLL4 alone induced the expression of *Csf2rb*, and TGFβ and DLL4 both individually induced *Tgfb1r*, with combined treatment having a greater effect (Fig. 8 G). These findings suggest that DLL4 signaling primes the differentiating cells to receive other signals from the alveolar niche, such as GM-CSF and TGFβ. TGFβ induced expression of *Rxra* and *Pparg* in GM-BMprogs (Fig. 8 H and Fig. S4 H), but treatment with DLL4 alone was unable to induce *Rxra* (Fig. 8 H), suggesting that *Rxra* expression induced by GM-CSF pre-treatment was already high and may have masked the DLL4-induced *Rxra* expression observed in M-BMprogs (Fig. 8, B and D). *Rxrb* expression in GM-BMprogs was induced only with DLL4 and not with TGFβ (Fig. 8 H). TGFβ and DLL4 were also required for the induction of many RXR target genes, including AM-specific genes such as *Cd11c* and *Siglecf* (Fig. S4 I), *Abca1* and *Tgfb1* (Fig. S4, J and K), and the AM-core genes *CD11a* and *Ucp3* (Fig. S4 L). Although TGFβ and DLL4 did not affect gene expression of *Notch2*, TGFβ induced expression of *Notch1*, and the combination of both signals produced a synergistic effect (Fig. S4 M).

These results provide evidence that GM-CSF, TGFβ, and DLL4 act as cooperative niche signals necessary for the induction of *Rxra* and *Rxrb* in differentiating macrophages and for the expression of key genes for AM identity, some of these being direct RXR targets.

### DLL4–NOTCH signaling is required for monocyte recruitment and AM maintenance

To investigate the importance of DLL4 signaling in AM differentiation, we used *Cdh5*^*Cre+ERT*^*Dll4*^*fl/fl*^ mice, which allowed us to delete *Dll4* in ECs after tamoxifen injection in 9-wk-old mice (Fig. 8 I), from which time the contribution of monocytes to the AM pool is exponential (Liu et al., 2019). 2 wk after DLL4 deletion, the AM population was reduced by twofold relative to *Dll4*^*fl/fl*^ mice (Fig. 8, J and K). *Cdh5*^*Cre+ERT*^*Dll4*^*fl/fl*^ mice had elevated numbers of circulating monocytes and blood neutrophils, excluding altered myelopoiesis as a possible cause of AM depletion (Fig. 8 L). To block NOTCH downstream signaling during monocyte recruitment and differentiation, we depleted the AM population in *Cd11c*^*Cre+*^*R26*^*LSL-DTR*^ mice by DT administration (Fig. 6 A) and then treated the mice with daily i.p. injections of the γ-secretase inhibitor LY-41157 (Sakai et al., 2019). Treatment with LY-41157 alone did not affect AM numbers (Fig. S4, N and O), as previously reported (Wong et al., 2004). FACS analysis at 1, 4, 7, and 10 days after treatment showed that, while *Cd11c*^*Cre+*^*R26*^*LSL-DTR*^ mice treated only with DT recovered lung AMs, mice treated with DT and LY-411575 failed to replenish the AM population from circulating monocytes (Fig. 8, M and N). These results led us to postulate that *in vivo* DLL4 directs the extravasation of circulating monocytes from the lung microcirculation to the alveolar lumen, where they receive GM-CSF and TGFβ signals from the cells of the niche. These niche-derived signals induce the expression of *Rxr* genes, which cooperatively promote the transcriptional and epigenetic changes that drive the differentiating monocytes to acquire final AM identity. To ascertain the extent to which our results reflect the human context, we examined publicly available scRNA-seq data from human lung (Travaglini et al., 2020). We identified >30 distinct cell types, encompassing epithelial cells, immune cells, interstitial cells, and cells of the lung vasculature (Fig. S4 P), and analyzed the expression of DLL4, GM-CSF, TGFβ, and their respective receptors. CellPhoneDB analysis showed DLL4 expression predominantly in the ECs of arteries and capillaries; moreover, consistent with our hypothesis, NOTCH2 was found in classical and nonclassical monocytes, as well as in other perivascular cells, including pericytes and ionocytes (Fig. S4 Q). ATII cells predominantly expressed GM-CSF, whereas the receptors for this cytokine were expressed on other cells, including AMs. The expression of TGFβ was widespread across various lung cell types, with their corresponding receptors also detected in a diverse range of cells that included macrophages. The analysis of human lung signals thus identified GM-CSF, TGFβ, DLL4, and their receptors as conserved features of the lung regulatory landscape and its preserved signaling niche.

### Cooperation between RBPJ, RXR, SMAD, and STAT5 orchestrates AM identity

**Figure 9.**
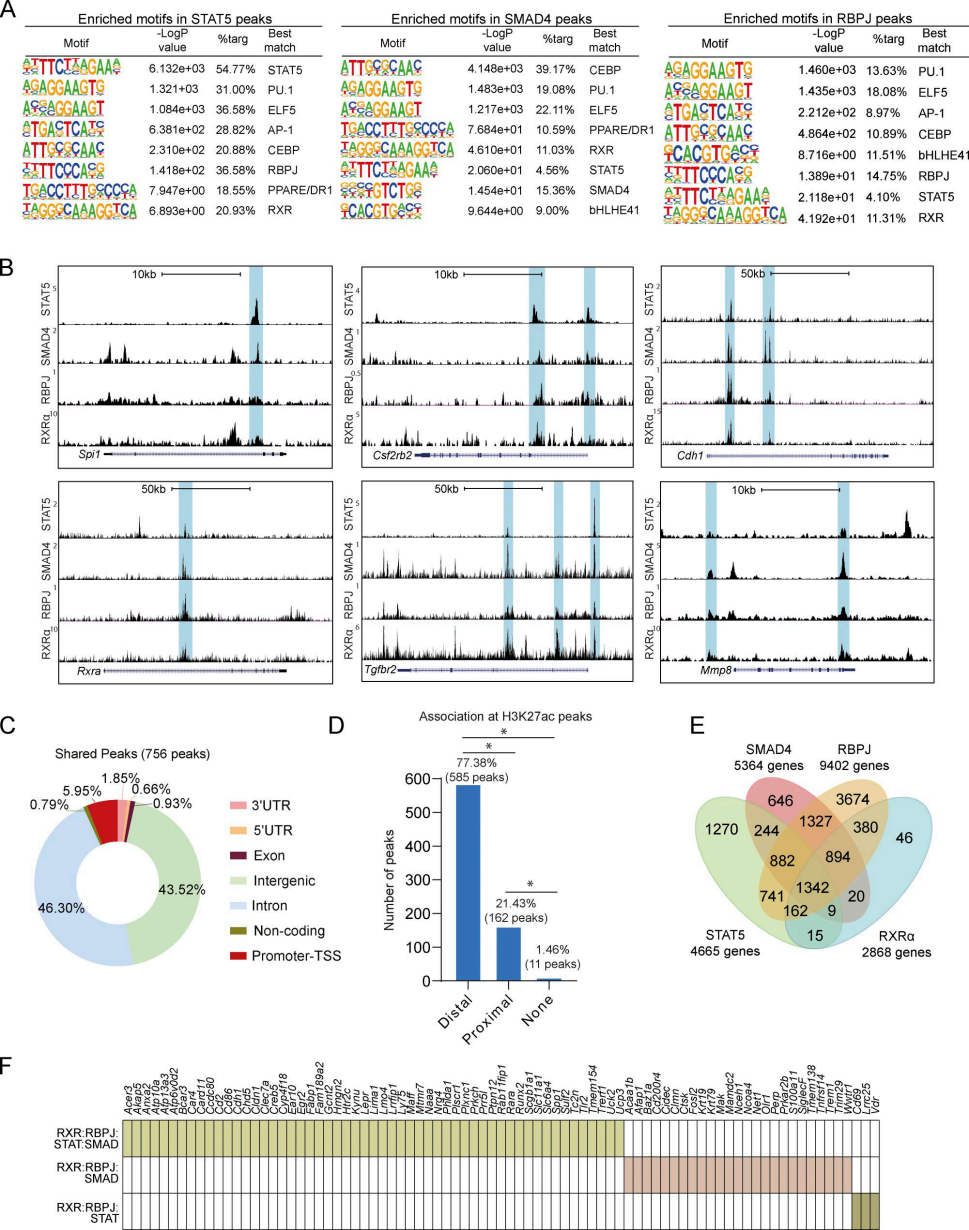
**AM identity depends on cooperation between RBPJ, RXR, SMAD, and STAT5. (A)** HOMER motif enrichment analysis for STAT5, SMAD4, and *in vivo* RBPJ peaks. **(B)** UCSC genome browser plots showing STAT5, SMAD4, RBPJ, and RXRα ChIP data peaks in macrophage-specific genes, *Rxra*, the GM-CSF and TGFβ receptor genes (*Csf2rb2* and *Tgfbr2*), and AM-core genes. The blue boxes indicate the areas with peaks identified in the different datasets. **(C)** Genomic feature distribution for 756 STAT5, SMAD4, RBPJ, and RXRα overlapping peaks. **(D)** Bar plot showing the association between 756 STAT5, SMAD4, RBPJ, and RXRα overlapping peaks and H3K27ac regions classified as proximal or distal according to their distance from the nearest gene. **(E)** Overlapping analysis of the collections of genes identified as nearest to STAT5, SMAD4, RBPJ, and RXRα regions detected by ChIP techniques. **(F)** Analysis of STAT5, SMAD4, RBPJ, and RXRα combinatorial regulation of annotated AM-core genes. *P ≤ 0.05; Test of equal or given proportions (prop.test function in R).

**Figure S5.**
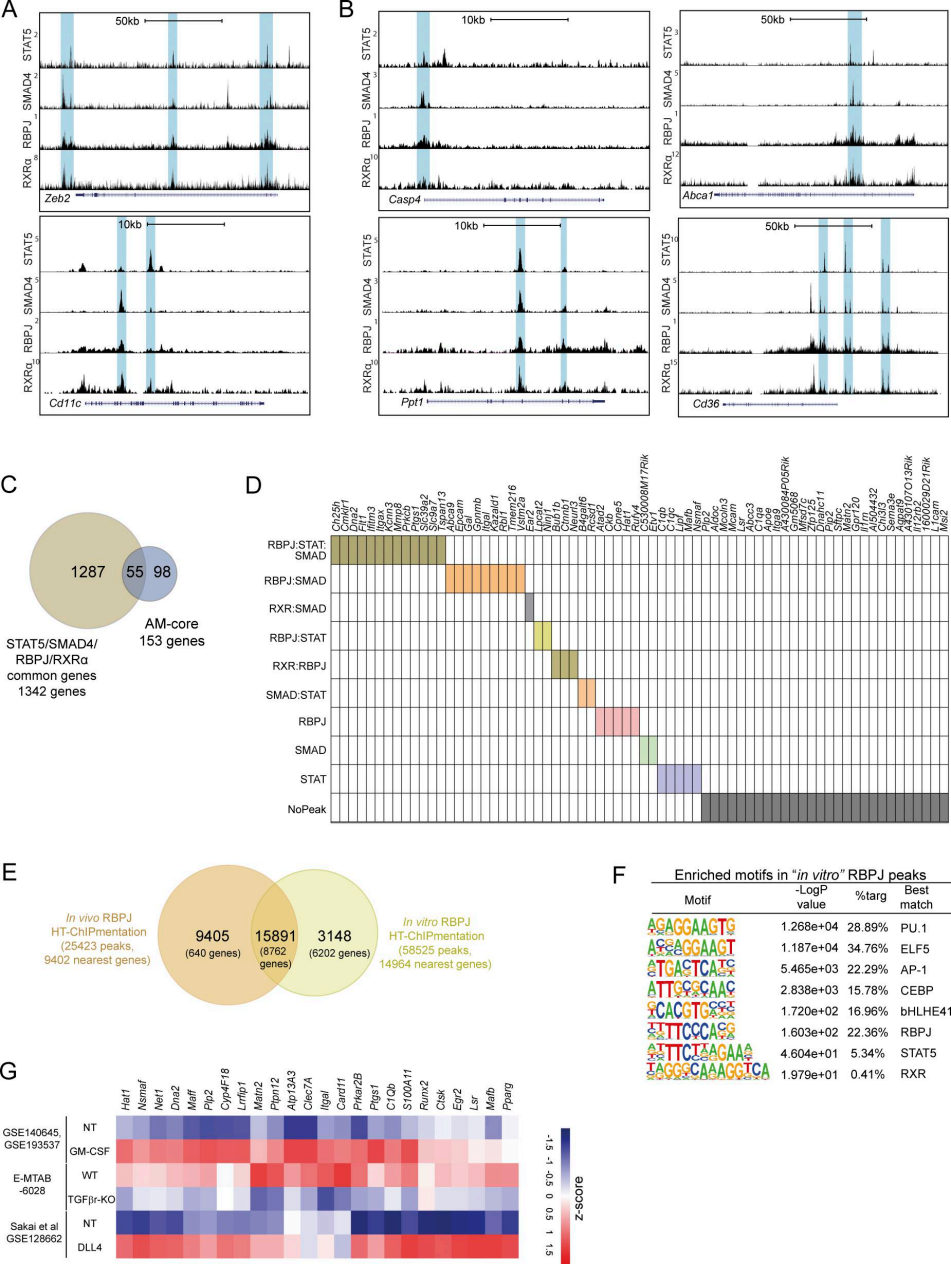
**RBPJ, RXR, SMAD, and STAT5 cooperate in the regulation of AM-core gene expression. (A and B)** UCSC genome browser plots showing STAT5, SMAD4, RBPJ, and RXRα ChIP data peaks in (A) *Zeb2* and *Cd11c* and (B) apoptosis- and lipid metabolism–related genes. The blue boxes indicate the areas with peaks identified in the different datasets. **(C)** Overlapping analysis between 1,342 genes identified as nearest to all STAT5, SMAD4, RBPJ, and RXRα ChIPmentation peak sets with AM-core genes. **(D)** STAT5, SMAD4, RBPJ, and RXRα combinatorial regulation of annotated AM-core genes. **(E)** Venn diagram showing overlap analysis between *in vivo* and *in vitro* RBPJ HT-ChIPmentation data in AMs. **(F)** HOMER motif enrichment analysis for *in vitro* RBPJ peaks. **(G)** Heatmap obtained using publicly available data, showing the expression values of AM-core genes upregulated after DLL4 or GM-CSF treatment and downregulated in TGFβr-KO AMs; NT, non-treated.

We next explored the potential interactions of RXR with the TFs acting downstream of GM-CSF, TGFβ, and DLL4: STAT5, SMAD4, and RBPJ, respectively. We used HT-ChIPmentation to generate a genome-wide profile of SMAD4 and RBPJ binding (Tables S6 and S7) and additionally accessed publicly available peak sets for STAT5 (GSM5625231, GM-CSF–treated cells) and RBPJ (GSE128662, DLL4-treated cells). HOMER motif enrichment analysis of STAT5, SMAD4, and RBPJ peaks showed enriched binding sites for key TFs for AM identity (ELF5, CEBP, PPARs, and bHLHE41), as well as the pan-macrophage LDTF PU.1 (Fig. 9 A). These peak sets were also enriched for motifs specific to RXRs and to the corresponding TF in each case (Fig. 9 A). To analyze the extent to which these factors collaborate in the determination of AM identity, we first explored the coinciding binding patterns of STAT5, SMAD4, RBPJ, and RXRα in the vicinities of the PU.1 gene *Spi1*, key AM-signaling elements for *Csf2rb2* and *Tgfbr2*, and the AM-core genes *Cdh1* and *Mmp8* (Fig. 9 B). Matching peaks were also found in the regulatory elements of the macrophage transcriptional modulator *Zeb2*, the AM-specific integrin *Cd11c*, the apoptosis-related genes *Casp4* and *Ppt1*, and the fatty-acid homeostasis-related genes *Abca1* and *Cd36* (Fig. S5, A and B). We found a set of 756 peaks that overlapped by at least one nucleotide across all four TF-binding patterns. Genome distribution analysis revealed that 5.95% of these shared peaks were located in promoters, 43.52% in intergenic regions, and 46.30% in intronic regions, indicating that the combined action of the four TFs in AMs takes place primarily through enhancers (Fig. 9 C). The intersection of the shared peaks with distance-discriminated H3K27ac showed an association with distal H3K27ac sites, confirming that cooperation among the four TFs occurs more frequently in enhancers (Fig. 9 D). The binding regions shared by STAT5, SMAD4, RBPJ, and RXR are preferentially and significantly located at distal regions (Fig. 9 D). Comparison of annotated genes for each experiment identified 1,342 overlapping genes for the four analyzed TFs (Fig. 9 E), including *Spi1*, *Csf2rb2*, and *Tgfbr2* and nearly 40% of the AM-core genes (Fig. S5 C). Analysis of the differential combination of the three other TFs with RXRα revealed that they were responsible for the regulation of 87% of the AM-core genes (Fig. 9 F and Fig. S5 D). For RBPJ, our *in vivo* data and the published *in vitro* dataset revealed similar binding patterns, suggesting that the *in vitro* data constitute a valid tool for defining the *in vivo* RBPJ-binding landscape (Fig. S5, E and F). Consistent with these findings, analysis of batch-corrected publicly available RNA expression data confirmed that TGFβ (E-MTAB-6028), GM-CSF (GSE140645 and GSE193537), and DLL4 (GSE128662) regulate the expression of a large set of AM genes (Fig. S5 G).

## Discussion

The results presented here demonstrate that alveolar GM-CSF and TGFβ, together with DLL4 expressed on the ECs of the alveolar vasculature, induce the expression in AM precursors of a set of TFs that cooperate with RXRα during the induction and regulation of mAM identity. Through these interactions, RXR signaling controls AM maturation, maintenance, and function. The absence of phenotype upon *Rxra* deletion in myeloid cells (Casanova-Acebes et al., 2020; Menendez-Gutierrez et al., 2015; Menendez-Gutierrez et al., 2023) indicates that RXR subtypes can compensate for each other in AMs. Recent work has suggested differential roles for these receptors in AMs (Wang et al., 2024); however, the limited transcriptional impact of RXRβ targeting and the low efficiency of CRISPR/Cas9 editing reported in that study raise concerns about incomplete RXRβ deletion, which may underlie the apparent discrepancies between studies. We therefore crossed *Rxrab*^fl/fl^ mice with mice harboring the driver *Cd11c*^*Cre*+^ to generate *Cd11c*^*Cre*+^*Rxrab*^fl/fl^ mice, with deletion of *Rxra* and *Rxrb* in the AM lineage. RXR deletion in this model disrupted lipid processing, resulting in lipotoxicity-induced cell death and a low number of mAMs, with altered gene expression. In addition, the lack of RXRs resulted in the accumulation of ImmAMs arrested at an intermediate stage of AM differentiation and characterized by high CD11b expression and failure to upregulate SiglecF. Supporting this hypothesis, competitive BM transplantation showed that RXR-deficient cells fail to contribute to the mAM pool, supporting a cell-intrinsic role for RXRs in AM maturation. A similar phenotype has been described in RXR-deficient LPMs (Casanova-Acebes et al., 2020), suggesting that RXRs might be an indispensable TF for embryo-derived TRMs.

Recent studies have challenged the classical view that AMs are exclusively embryo-derived, suggesting that they can also be replenished by BM-derived monocytes. When BM-derived monocytes infiltrate tissues, they undergo transcriptional changes resulting in phenocopy of embryo-derived macrophages (Bain et al., 2016; Cain et al., 2013). This maturation process could be also impaired in the absence of RXRs. Further dissection of this phenomenon would require cell-tracking assays or mouse models allowing the apoptotic and remaining embryo-derived AMs to be distinguished from the incoming BM cells.

Our transcriptomic data indicate that RXR-deficient AMs downregulate lipid metabolic processes and have an enhanced rate of cell death. The RNA-seq data revealed that RXRs control the expression of many AM-core genes and TFs important for AM identity. Furthermore, lack of RXRs also disrupted expression of the receptors for two key instructive signals of AM identity, GM-CSF and TGFβ (Guilliams et al., 2013; Yu et al., 2017). Unable to receive these instructive niche signals, RXR-deficient AM precursors do not undergo the required definitive transcriptional changes. This could account for the observed immature phenotype of RXR-deficient AMs. Our findings also show that RXRs are needed to maintain a chromatin landscape that allows AM-identity TFs to bind and function. Deleting RXRs in AMs results in a general loss of chromatin accessibility without a reorganization of specific genomic sites. Interestingly, studies with RXR-deficient LPMs showed that there were no changes in chromatin openness and that open peaks were rearranged toward distal regions (Casanova-Acebes et al., 2020). Other studies have also shown that the lack of RXR in dormant long-term hematopoietic stem cells results in chromatin opening and a reorganization of promoters and intergenic regions (Menendez-Gutierrez et al., 2023). These differing patterns of altered chromatin accessibility upon RXR deletion might suggest that RXRs control chromatin structure in a cell type–specific manner.

The results of our combined transcriptomic and HT-ChIPmentation analysis highlight the pivotal role of RXRs in establishing AM population and identity, demonstrating that they control the expression of LDTFs that regulate the macrophage-core program, including *Spi1*, *Cebpb*, and *Runx1*, as well as other important AM TFs, such as *Pparg* and *Bhlhe40/41*, that drive the functional specialization of AMs in the alveolus. The functional identity of TRMs develops through the activation of specific functional modules by distinct TFs, shaping and complementing the core macrophage program to specialize each macrophage subset (T'Jonck et al., 2018). In this regard, the large downstream TF network governed by RXRs in AMs, together with the regulation of key identity and function genes, explains the nonfunctional immature phenotype observed upon their deletion. The motif enrichment analysis of the RXR peaks indicates that RXRs cooperate with other nuclear receptors, including PPARs. Interestingly, PPARγ-deficient lungs exhibit reduced numbers of AMs in adulthood and during development, and these cells have an immature phenotype and accumulate lipids (Schneider et al., 2014). The similar transcriptomic profiles of RXR and PPARγ, together with their overlapping binding site occupancy in the HT-ChIPmentation analysis, provide strong evidence for a shared gene regulatory network driven by RXR–PPARγ heterodimerization in AMs. This suggests that the phenotype of RXR-deficient AMs might be explained by the loss of heterodimerization with PPARγ. However, RXRs also form heterodimers with other nuclear receptors expressed in AMs, such as RAR or LXR (Gautier et al., 2012), as well as RXR homodimers, and possible roles for these pairings cannot be discarded. Although the survival of the macrophage in its niche often depends on the activation of functional specialization modules, this specialization may not be significant in steady-state conditions (T'Jonck et al., 2018). Further investigation of the importance of RXRs in the functional specialization of AMs would therefore require the use of damage and inflammation models.

The induced depletion of AMs showed that circulating monocytes are recruited to the lung and rapidly downregulate monocyte-specific genes while initiating the expression of AM-specific genes and the receptors for the alveolar cytokines GM-CSF and TGFβ. In this transcriptional shift, the expression of RXRs is promptly induced, supporting the idea that RXRs are key TFs for AM differentiation and identity acquisition. The contribution of circulating monocytes to the AM population requires DLL4, as demonstrated both by the inducible deletion of DLL4 in 9-wk-old mice and by the blockade of the NOTCH signaling pathway in AM-depleted lungs. Previous studies in TRMs indicated that AMs are maintained locally, with a minimal contribution from BM-derived monocytes (Hashimoto et al., 2013; Hu and Christman, 2019; Ginhoux and Guilliams, 2016). However, our observations show that AMs are not a fully self-sufficient population and that they receive a time-dependent contribution from circulating monocytes. Consistent with our results, published fate mapping studies show that age-dependent lung growth indeed requires a contribution to the AM pool from BM cells (Joshi et al., 2018), especially from 8 wk of age (Gomez Perdiguero et al., 2015; Liu et al., 2019). Our results demonstrate that DLL4, through its collaboration with GM-CSF and TGFβ, is necessary for the complete induction of *Rxra* and *Rxrb*. Importantly, the receptors of GM-CSF and TGFβ are targets of RXRs, implying that DLL4 signaling triggers an RXR-dependent positive feedback loop. The AM population was completely reconstituted within the first 10 days after treatment of *Cd11c*^*Cre+*^*R26*^*LSL-DTR*^ mice with DT. However, AM reconstitution was blocked in mice treated with the Notch pathway inhibitor LY-411575. This suggests that at least in this time frame, the alveoli are repopulated predominantly by recruited monocytes and that the contribution to the AM pool from other cells, such as IMs, is not significant. The induction of a specific macrophage transcriptional landscape is dependent on the sensing of specific niche-derived signals (T'Jonck et al., 2018). For example, PMs transplanted into the alveolar niche receive lung-derived signals that induce the loss of peritoneal markers and the acquisition of those characteristic of AM identity (Lavin et al., 2014). Moreover, AMs extracted from the lungs change their phenotype even in the presence of GM-CSF, although phenotype is restored upon reinsertion into the alveolar niche (Subramanian et al., 2022). In this regard, our results demonstrate that not only the instructive GM-CSF and TGFβ but also endothelial cell–expressed DLL4 act as cooperative lung signals through RXRs to shape the epigenetic and transcriptional footprint of AMs in recruited and repopulating cells and might be essential for maintenance of the AM phenotype *ex vivo*. The cross validation between our *in vitro* studies and the human scRNA-Seq data underscores the physiological relevance of these conserved signaling pathways to both mouse and human lung biology. Our discovery paves the way for future translational research, offering a promising field for the development of targeted interventions and therapeutic strategies. Taken together, our results demonstrate that DLL4 acts as an initial signal that permits the recruitment of differentiating monocytes to the lung and triggers the RXR-dependent program to receive AM key niche cytokines.

Our results further highlight the importance of tissue signals as determinants of TRM identity (Guilliams et al., 2020). We provide evidence that RXRs interact collaboratively with other TFs to specify the AM regulatory landscape and the core AM gene network. Our comprehensive analysis, including ChIP sequencing (ChIP-seq) and motif-enrichment analysis, establishes a hierarchical relationship among STAT5, SMAD4, RBPJ, and RXR. RBPJ has binding peaks at the *Rxra* and *Rxrb* loci, indicating direct regulation of RXRs. STAT5 and SMAD4 also have peaks at the *Rxra* locus, confirming the role of GM-CSF and TGFβ in the induction of RXR expression. The analysis of motif enrichments in the peaks of each of these TFs confirmed the presence of binding motifs for the other TFs in the set, reaffirming the cooperation between them. The colocalization of these proteins in the vicinity of genes such as *Spi1* and *Zeb2* suggests a mechanism in which the collaboration of these TFs creates positive feedback loops that reinforce the expression of key LDTFs for macrophage differentiation. Moreover, the importance of cooperation between STAT5, SMAD4, RBPJ, and RXRs is underscored by the existence of common peaks for these TFs in genes involved in alveolar niche signaling and AM-core gene expression. Although our study focuses on these TFs, it would be premature to rule out possible roles in AM-identity acquisition of other TFs such as CEBP or ELF5, TF repressors such as bHLHEs, or other nuclear receptors, including PPARs. Together with the results of the AM depletion experiments, the ChIP-seq and motif-enrichment analysis support a model in which interaction with ECs in the alveolar vasculature induces infiltrating monocytes to extravasate to the alveoli, where they receive coordinated niche signals that rapidly upregulate the expression of RXRs. In turn, RXRs act as master TFs and collaborate with STAT5, SMAD4, and RBPJ to regulate the expression of AM-core genes. Thus, in this model, DLL4 from the endothelium and TGFβ and GM-CSF from the alveolar niche trigger a process of specialization in which the infiltrating monocyte acquires the phenotypic and functional characteristics that define AM identity.

The importance of macrophage RXR expression has been substantiated in several pathological contexts (Lefebvre et al., 2010; Perez et al., 2012; Rőszer et al., 2013). Future studies are warranted to further explore the signaling pathways and TF cooperation elucidated here. Furthermore, elucidation of the specific RXR ligand in AMs holds promise for the development of treatments for diseases associated with AM dysfunction, including alveolar proteinosis, cancer, and COVID-19. Our approach, encompassing *in vitro*, *in vivo*, and genome-wide analyses, unveils the intricate regulation, organization, and functional importance of TFs in response to described instructive niche signals. Moreover, we provide insight into the mechanism underlying the differentiation of circulating monocytes into mAMs, with a particular emphasis on the indispensable role of the nuclear receptor RXR in determining AM identity.

## Materials and methods

### Mice

All mice used in this study (both male and female) had the C57BL/6J background and were housed (from two to five animals per cage) under a 12 h light/dark cycle at a constant temperature of 23°C with *ad libitum* access to food and water. All experiments were performed according to local ethical guidelines and were approved by the Animal Subjects Committee of the Instituto de Salud Carlos III (Madrid, Spain) in accordance with EU Directive 86/609/EEC. The mouse models used in this study were *LysM*^*Cre+*^ (Clausen et al., 1999), *Cd11c*^*Cre+*^ (Brocker et al., 1997), and *Vav*^*iCre*+^ (de Boer et al., 2003). To conditionally delete *Rxra* and *Rxrb*, these lines were crossed with *Rxrab*^*fl/fl*^ mice, generating *LysM*^*Cre+*^*Rxra*^*fl/fl*^*Rxrb*^*fl/fl*^ mice (*LysM*^*Cre+*^*Rxrab*^*fl/fl*^), *Cd11c*^*Cre+*^*Rxra*^*fl/fl*^*Rxrb*^*fl/fl*^ mice (*Cd11c*^*Cre+*^*Rxrab*^*fl/fl*^), and *Vav*^*iCre*+^*Rxra*^*fl/fl*^*Rxrb*^*fl/fl*^ mice (*Vav*^*iCre*+^*Rxrab*^*fl/fl*^) (Casanova-Acebes et al., 2020; Menendez-Gutierrez et al., 2023). *Rxrab*^*fl/fl*^ littermates were used as controls. *Rxrb*^*fl/fl*^ mice were kindly provided by D. Metzger (Institute of Genetics, Molecular and Cellular Biology, Illkirch-Graffenstaden, France)*.* To conditionally delete *Dll4* in ECs, we crossed Tg(*Cdh5-CreERT2*) mice with *Dll4*^fl/fl^ mice (Fernández-Chacón et al., 2023). To induce CreERT2 activity in adult mice, mice received i.p. injections of 2 mg tamoxifen dissolved in corn oil on three consecutive days, as previously described (Fernández-Chacón et al., 2023), and were analyzed 2 wk after the last injection. Littermates carrying the *Dll4*^*fl/fl*^ allele but no Cre transgene also received tamoxifen injections and were used as controls. To characterize the recruitment of monocytes upon AM ablation, we crossed *Cd11c*^*Cre+*^ mice with *R26*^*LSL-DTR*^ mice (Buch et al., 2005). The resulting *Cd11c*^*Cre+*^*R26*^*LSL-DTR*^ mice were intratracheally instilled with a saline solution of 100 ng unnicked *Corynebacterium* DT (Sigma-Aldrich). Male and female mice were studied from E18.5 to 9–11 wk of age. Before tissue harvesting, embryos and neonatal mice from DAB0-DAB5 were sacrificed by decapitation. For DAB10-11 wk-old and older mice, mice were sacrificed by asphyxiation in a carbon dioxide (CO_2_) chamber.

Mice were genotyped by PCR with the following primers: P1 and P2 for floxed *Rxra* (800 bp floxed band); XO141 and WS55 for floxed *Rxrb* (270 bp floxed band); Cre1 and Cre2 for Cre in Cre-baring models (450 bp); DLL4fl-F and DLL4fl-R primers for the floxed Dll4 allele (500 bp floxed band). The Cre allele in *Cdh5*^*Cre+ERT*^ mice was directly detected with CreM-F and CreM-R primers (550 bp). Primer sequences are provided in Table S8.

### Cell isolation

Cell suspensions were obtained from lungs, BAL, and PB. For BAL, the alveolar cavities of adult mice were washed seven times with 1 ml of sterile PBS containing 1% FBS and 10 mM EDTA. PB samples (20–100 μl) were obtained by puncture of the facial vein and subsequent erythrocyte lysis for 5 min with 1 ml of RBC Lysis Buffer (eBioscience). For lung homogenates, lungs were chopped finely and digested at 37°C for 30 min with 0.8 mg/ml collagenase type IV (Sigma-Aldrich). The lung homogenates were then syringed through an 18-G needle 15 times, and the resulting cell suspension was passed through a 100-µM nylon filter. Any remaining RBCs were lysed by addition of 500 μl RBC Lysis Buffer and incubation for 3 min. After lysis, cell preparations were centrifuged at 1,500 rpm for 5 min, and pelleted cells were resuspended for subsequent assays.

### Flow cytometry

Cell suspensions (up to 10 × 10^6^ cells) were blocked with an anti-CD16/32 antibody (BioLegend) and then stained for 30 min at 4°C with appropriate antibodies (full list in Table S9). Unbound antibodies were washed off with 500 μl of 0.5% BSA in PBS. Cell suspensions were prepared for flow cytometry analysis in 250 μl 0.5% BSA/PBS containing 10 µg/ml DAPI. For apoptosis studies, cells were labeled with anti-Annexin-V antibody (BioLegend) in Annexin-V buffer (BD Biosciences). After washing, cells were stained with DAPI and analyzed by FACS within 1 h after labeling. For the study of BrdU incorporation in proliferating cells, mice received seven daily i.p. injections of 1 mg BrdU (BD Biosciences). Lungs were harvested 24 h after the last BrdU injection. Incorporated BrdU was detected according to the FITC BrdU flow kit instruction manual (BD Biosciences). For Ki-67 assay, cells were fixed and permeabilized with the Foxp3 staining buffer set (Thermo Fischer Scientific) and incubated with anti-Ki-67 antibody (BioLegend) and DAPI. For BODIPY staining, cells were incubated for 20 min at 4°C with BODIPY493/503. Flow cytometry was performed with Spectral (SONY), Fortessa (BD, Biosciences), and Canto 3L (BD, Biosciences) analyzers, and data analysis (including t-SNE representation) was performed with FlowJo 10.4.2 software. For sorting, we used customized FACSAria II and FACSAria Fusion flow cytometers (BD Biosciences) or a customized SY3200 Cell Sorter (SONY).

### Histological analysis

Lungs were fixed for 48 h in 4% paraformaldehyde (PFA). Lung preparations were incubated in 70% ethanol and subsequently embedded in paraffin for sectioning Microtome sections (5 μm) were cut and stained with H&E or processed for elastic Van Gieson staining. Tissue sections were scanned using NanoZoomer-2.0RS C110730, and images were analyzed with the NDP.2 viewer.

### Oil Red O staining

Cell suspensions obtained from BAL were cytospun at 600 rpm for 5 min at room temperature (RT). Cytospin preparations were washed with PBS, and cells were fixed for 24 h with 4% PFA. Cells were washed once with 60% isopropanol and left to dry overnight. Preparations were then stained with 500 μl Oil Red O for 15 min at RT and washed once with distilled water. Preparations were then incubated in hematoxylin for 30 s and received a final distilled water wash. NanoZoomer-2.0RS C110730 was used to scan tissue sections and later analyzed using the NDP.2 viewer.

### Quantification of BAL proteins and phospholipids

Cell-free BAL supernatant was obtained by centrifugation for 5 min at 700 *g* and 4°C. Total BAL protein concentration was measured in cell-free BAL supernatants by the Lowry method modified by the addition of SDS (Casals et al., 1998). Lipids were extracted with chloroform and methanol (Casals et al., 1989; Casals et al., 1998). BAL phospholipids were quantified by phosphorus analysis, and phosphatidylcholine concentration was measured with enzymatic methods (Spinreact) (Casals et al., 2003).

### Western blot analysis of surfactant proteins

The four surfactant proteins SP-A, -B, -C, and -D were detected by western blot, as described (Casals et al., 2003). SP-A and SP-B were detected using anti–human-SP-A and anti-porcine SP-B polyclonal antibodies prepared in our laboratory. SP-C was detected using an anti-recombinant human SP-C polyclonal antibody kindly provided by Nycomed Pharma. SP-D was detected with an anti-SP-D mAb (1A10A9; Seven Hills Bioreagents). Bound primary antibodies were detected by incubation of blots with appropriate secondary antibodies (Sigma-Aldrich) and revealed with chemiluminescence (Amersham ECL Select, GE Healthcare). Protein bands were quantified by densitometry using Quantity One software (Bio-Rad Laboratories).

### Competitive BM transplant

CD45.1^+^CD45.2^+^ recipient mice were lethally irradiated with two doses of 5.5 Gy, with doses separated by a 4-h interval. Mice were reconstituted 1 h after the final radiation dose with 2.5 × 10^6^ BM cells from *Rxrab*^*fl/fl*^ or *Cd11c*^*Cre+*^*Rxrab*^*fl/fl*^ CD45.2^+^ mice together with an equal number of BM cells from CD45.1^+^ WT mice. Chimerism was assessed in PB 4, 8, or 16 wk after reconstitution. Lung cells were collected and analyzed at the experimental endpoint.

### qPCR

Total RNA was isolated with Trizol (Sigma-Aldrich) and Max Extract High Density tubes (Qiagen) or with the Arcturus PicoPure RNA Isolation Kit (Thermo Fischer Scientific). Transcripts were quantified using the AB7900-FAST-384 system with a two-step reverse-transcription qPCR process. Gene expression values were normalized to the housekeeping genes 36b4 and cyclophilin and presented as relative mRNA levels or fold differences compared with littermate control mice. Data were analyzed using qBASE software (Biogazelle). Primer sequences are listed in Table S8.

### Bulk RNA-seq

Total RNA was extracted from sorted mAMs and ImmAMs from BAL (40,000 cells). Cells were lysed in RLT buffer, and RNA was extracted according to the RNeasy Mini Kit protocol (Qiagen). RNA quality and concentration were measured by RNA 6000 Nano Assay (Agilent Technologies) in a Bioanalyzer 2100 instrument. RNA-seq libraries were prepared with the NEBNext Ultra II Directional RNA Library preparation kit (New England Biolabs). Sequencing was performed in a NextSeq 2000 system (Illumina) to generate 60-base single-end reads. Sequencing reads were preprocessed with a pipeline that used FastQC to assess read quality and Cutadapt to trim sequencing reads, eliminate Illumina adapter contaminations, and discard reads <30 bp. The resulting reads were mapped against reference transcriptome GRCm38_99 and quantified using RSEM (Li and Dewey, 2011). The percentage of reads participating in at least one reported alignment was ∼84%. Expected expression counts calculated with RSEM were then processed with an analysis pipeline that used the Bioconductor package Limma (Ritchie et al., 2015) to normalize (by the TMM method) and to test differential expression, taking into account only those genes expressed with at least 1 count per million in at least three samples. Changes in gene expression were considered significant if associated with a Benjamini and Hochberg p-adj <0.05. Functional enrichment analyses were performed with PANTHER (http://pantherdb.org/), QIAGEN’s IPA and GSEA (https://www.gsea-msigdb.org/gsea/index.jsp). Significantly enriched functions or regulators were identified by applying Benjamini and Hochberg p-adj thresholds <0.05 for PANTHER and IPA and FDR q_value <0.25 for GSEA. RNA-seq quality data are provided in Table S10.

### ATAC-seq

Epigenomic profiling of chromatin accessibility was assessed by ATAC-seq as described by Buenrostro et al. (2015). The protocol was as described by Menendez-Gutierrez et al. (2023). AMs from BAL (20,000 per replicate) were centrifuged at 500 *g* for 20 min at 4°C. Cells were lysed (10 mM Tris-HCl, pH 7.4, 10 mM MgCl_2_, and 0.1% IGEPAL CA-630), and nuclei were pelleted. For the transposase reaction, 20,000 isolated nuclei were incubated for 30 min at 37°C with 2.5 μl per sample of Tn5 from the Nextera DNA Library Preparation Kit (Illumina). SDS was then added to 1% vol/vol, and the incubation continued for 5 min at RT. DNA was purified with the ChIP DNA Clean & Concentrator kit (Zymo). PCR amplification and barcoding were done with the primers described by Buenrostro et al. (2015). PCR reactions included 11 μl NEB2 × PCR Mix (New England Biolabs), 10 μl tagmented DNA, 0.2 μl primer Ad_ no Mx (forward), and 0.2 μl barcoded reverse primer (Ad_2.1 to Ad_2.8) (primer sequences are listed in Table S8). The first PCR program was 72°C for 5 min; 98°C for 30 s; and 5 cycles of 98°C for 10 s, 63°C for 30 s, and 72°C for 1 min, with the temperature reducing to 4°C after the final cycle. DNA was size selected with 0.65 × volume SPRI beads (Agencourt AMPure, Beckman Coulter) and cleaned with 1.5 × SPRI beads. The second PCR program was 98°C for 30 s, followed by four cycles of 98°C for 10 s, 63°C for 30 s, and 72°C for 1 min. Libraries were purified with 1.5 × SPRI beads, concentration was measured with the Qbit dsDNA HS Assay kit (Thermo Fischer Scientific), and fragment profiles were analyzed with the Bioanalyzer DNA High Sensitivity Kit (Agilent). Libraries were sequenced in 2 × 50 HiSeq 3000 instrument (Illumina), with an average of 25 million paired-end reads per sample.

Reads were analyzed as in Menendez-Gutierrez et al. (2023). After merging of reads corresponding to the same sample, Cutadapt version 1.7.1 was used to trim adapters (Martin, 2011). Trimmed paired-end reads were aligned to the mm10 mouse reference genome using Bowtie2 version 4.1.2 (Langmead and Salzberg, 2012) with settings “-X 2000 -very-sensitive” in paired-end mode. Duplicates were marked with PICARD tools (http://picard.sourceforge.net). Alignments were filtered with samtools version 0.1.18 (Li et al., 2009) to keep only non-duplicated, paired, and properly mapped reads, as well as by alignment quality (>Q30). Peak calling was performed with MACS3 version 3.0.0a5 (Zhang et al., 2008) with parameters “-nomodel -shift −100 -extsize 200 -qval 0.05.” A consensus peak set of peaks detected in at least two samples was generated using DiffBind R package version 2.6.6 (function dba.counts) (Stark and Brown, 2011; Ross-Innes et al., 2012). EdgeR version 3.20.9 (Robinson et al., 2010) was used to perform differential accessibility using DiffBind functions (dba.analyze). Regions with differential accessibility were defined by FDR <0.05. Peak annotation, including the identification of the nearest gene TSS, was attained with the command annotatePeaks.pl in HOMER version 4.10.3 (Heinz et al., 2010). TF motif enrichment analyses were performed with the HOMER command findMotifsGenome.pl. RXR-deficient model-specific peaks with increased accessibility were compared with *Rxrab*^fl/fl^-specific peaks and vice versa*,* and genomic heatmaps were created with deepTools2 [62] via Galaxy (https://usegalaxy.org/). ATAC-seq quality data are provided in Table S10.

### ChIP and sequencing with HT-ChIPmentation

Binding of RXR, SMAD4, RBPJ, PPARγ, and H3K27Ac was assessed by HT-ChIPmentation in AMs isolated from BAL (100,000–200,000 cells) of 9-wk-old WT C57BL/6J mice, following the protocol of Gustafsson et al. (2019). Briefly, after cell labeling for flow cytometry, cells were fixed in 2% FBS/PBS containing 1% formaldehyde for 10 min at RT. Fixation was stopped by adding 1 volume of 1 M glycine and incubating for an additional 10 min at RT. Cells were centrifuged at 2,000 *g* for 10 min at 4°C, then washed with 1 volume of 0.1 M glycine and centrifuged again. Cells were sorted, resuspended in 100 μl lysis buffer (50 mM Tris/HCL, pH 8.0, 0.5% SDS, and 10 mM EDTA), supplemented with complete EDTA-free protease inhibitor (Roche), and stored at −80°C overnight. The next day, cell suspensions were kept at 4°C for >20 min prior to sonication. For RXR, SMAD4, and H3K27ac, samples were placed in 130-μl Covaris AFA fiber tubes and sonicated with a Covaris S220 sonicator for 12 min (duty 5%, cycles per burst: 200, and PIP 140). For RBPJ, PPARγ, and H3K27ac, samples were transferred to eight-tube AFA bead strips and sonicated for 15 min using a Covaris E220 sonicator (duty 5%, cycles per burst: 200, and PIP 140). After sonication, SDS was neutralized by adding 6 μl 20% Triton X-100 (final concentration ≈1%), and 2 μl 50× protease inhibitors were added. Lysed cell suspensions were mixed with 50 μl ChIPmentation dilution buffer (50 mM Tris/HCL, pH 8.0, 225 mM NaCl, 0.15% Na deoxycholate [DOC], and 1.5% Triton X 100) containing previously coupled 10 μl protein G dynabeads (Thermo Fischer Scientific and Invitrogen) with 4 μg anti-RXRα (Proteintech), 3 μg anti-SMAD4 (Cell Signaling), 0.5 μg anti-RBPJ (Abcam), 1 μg anti-PPARγ (Diagenode), or 0.5 μg anti-H3K27ac (Active Motif, Cell Signaling). The mixture was incubated overnight at 4°C with rotation. Bead-bound chromatin was washed sequentially once each with the following buffers using the SMARTer-Seq Magnetic Separator for PCR strips (Takara Bio): wash buffer I (50 mM Tris/HCL, pH 8.0, 150 mM NaCl, 0.1% SDS, 0.1% NaDOC, 1% Triton X 100, and 1 mM EDTA, pH 8.0), wash buffer II (50 mM Tris/HCL, pH 8.0, 500 mM NaCl, 0.1% SDS, 0.1% NaDOC, 1% Triton X 100, and 1 mM EDTA, pH 8.0), and wash buffer III (10 mM Tris/HCL, pH 8.0, 250 mM LiCl, 0.5% NP-40, 0.5% NaDOC, and 1 mM EDTA, pH 8.0). These steps were followed by two washes with TE (10 mM Tris/HCl and 1 mM EDTA, pH 8.0) and another two washes with cold Tris/HCl 10 mM, pH 8.0. Washed bead-bound chromatin was resuspended in 30 μl 1× tagmentation buffer +1 μl Tagment DNA enzyme (Illumina) and incubated for 10 min at 37°C. Samples were then washed twice with wash buffer I, and the bead-bound tagmented chromatin was resuspended in a solution combining 25 μl NEB2 × PCR Mix (New England Biolabs), 23 μl distilled water, and the primers described by Buenrostro et al. (2015) for barcoding: 1 μl Ad_no Mx forward nonbarcoded primer + 1 μl barcoded primers (Ad_2.1 to Ad_2.7 sequences are listed in Table S8). The PCR program was 72°C for 5 min; 95°C for 5 min; 98°C for 30 s; and 11 cycles of 98°C for 10 s, 63°C for 30 s, and 72°C for 3 min, with the temperature reducing to 4°C after the final cycle. After library preparation, DNA was size selected with SPRI beads (Agencourt AMPure, Beckman Coulter) in a 1:1 proportion. Library DNA was released from SPRI beads by incubation with 14 μl distilled water. Sequencing reads were processed as described for ATAC-seq, but using input sample controls and default parameters for peak calling with MACS3, including the use of “-q 0.05” as the FDR cutoff. Genomic heatmaps were generated with deepTools2 (Ramírez et al., 2016). Other data manipulations and graphical representations (bar and volcano plots) were produced with R and GraphPad Prism version 9. Quality data for HT-ChIPmentation sequencing are provided in Table S10.

### CellPhoneDB ligand–receptor analysis

To infer cell–cell communication in the lung, we used publicly available mouse and human lung scRNA-seq data (GSE119228 and EGAS00001004344, respectively) (Cohen et al., 2018; Travaglini et al., 2020). For mouse data, we reanalyzed the experiment from the raw counts provided using the R packages scater (McCarthy et al., 2017) and Seurat (Hao et al., 2021). First, we selected high quality cells based on the following criteria: annotated as a single cell per well, between 500 and 20,000 counts, over 250 genes detected, below 15% MT content, below 25% counts on ERCC control probes, below 65% counts in the top 50 expressed genes, and below 0.1% counts in hemoglobin genes. Resulting cells were clustered with the following main parametrization: top 1,000 variable genes, 10 principal components for neighbor graph and umap generation, and 0.5 as clustering resolution. Resulting clusters were manually annotated according to marker expression: neumocyte type 1 (AT1) (*Akap5*), neumocyte type 2 (AT2) (*Lamp3*), B cells (*Cd19*), basophils (*Cd69*), ciliated cells (*Sec14l3*), endothelial cells (*Cdh5*), eosinophils (*Csf1r* and *Siglecf*), fibroblasts (*Col1a2* and *Mfap4*), ILC (*Rora*), macrophages (*Ear2*), mesenchymal stromal cells (*Ly6a*), monocytes (*F13a1*), neutrophils (*S1009a* and *Retnlg*), NK cells (*Ccl5*), other epithelial cells (*Igf2*), pericytes (*Col1a2*, *Gucy1a3*), smooth muscle cells (*Enpp2*), and T cells (*Trbc2*). Then, batches from MER1 experiment were removed, and the remaining data were analyzed for curated ligand–receptor pairs using CellPhoneDB version 2.0 statistical_analysis method as described in the published protocol with the following parameters: database v4.0.0, result-precision 7, threshold 0.005, and 500 iterations (Efremova et al., 2020). Selected significant interactions with monocytes and macrophages as targets were represented using R tools. (Some CellPhone version 4 database interactions were inverted to reflect the receptor nature of the molecule as target and ligand as source). For human data, we selected the normal lung datasets (Seurat objects) from patients 1–3 and integrated them using the reciprocal PCA method implemented in Seurat. Some cell labels from similar cell types were fused to simplify the analysis. Cell communication was inferred using LIANA version 0.1.12 (Dimitrov et al., 2022). The liana_wrap function was run with default parameters, and the output was aggregated using the liana_aggregate function. Results were filtered using an aggregate_rank <0.01, and LIANA plot functions were used to display them. Ligand–receptor expression plots were generated using normalized data from the integrated Seurat object.

### *In vitro* treatments

Cells were cultured at 37°C and 5% CO_2_ in complete RPMI supplemented with 10% FBS and 1% penicillin + streptomycin. To obtain BM progenitor cultures, 10 million flushed BM cells from C57BL/6J mice were cultured for 24 h in non-tissue culture dishes. Progenitors were then differentiated for 3 days with 20 ng/ml M-CSF to generate M-CSF–derived BM progenitors (M-BMprogs) or with 30 ng/ml GM-CSF to generate GM-CSF–derived BM progenitors (GM-BMprogs). After differentiation, adherent cells were re-cultured in 24-well culture plates as follows: 0.5 × 10^6^ cells per well were cultured in 0.5 ml fresh medium supplemented with 20 ng/ml M-CSF or 30 ng/ml GM-CSF plus 0.5 ml of the corresponding previous cell culture medium. During replating, the following stimuli were added alone or in the required combination for 24 h: DLL4 (1 μg/ml, R&D systems), GM-CSF (10 ng/ml, PeproTech), TGF-β1 (10 ng/ml, Abcam), TNFα (10 ng/ml, Sigma-Aldrich), M-CSF (10 ng/ml, PeproTech), IL-4 (10 ng/ml, R&D systems), IL-6 (10 ng/ml, Chemicon International), IL-1 (10 ng/ml, PeproTech), IL-33 (30 ng/ml, PeproTech), CCL3 (10 ng/ml, PeproTech), CCL5 (20 ng/ml, PeproTech), and FGF2 (50 ng/ml, Thermo Fischer Scientific). For DLL4 stimulation, culture-treated plates were previously coated with 1 μg/ml DLL4 for 1 h at RT. A similar pre-coating procedure was used for fibronectin (5 μg/ml, Sigma-Aldrich).

### DLL4 signaling inhibition

To induce AM ablation, *Cd11c*^*Cre+*^*R26*^*LSL-DTR*^ mice were intratracheally instilled with PBS containing 100 ng unnicked *Corynebacterium* DT (Sigma-Aldrich). When required, mice subsequently received daily i.p. injections of 10 mg/kg LY-411575 (MedChem) dissolved in 1:9 DMSO:corn oil for 1, 4, 7, or 10 days. Lungs were then isolated and processed, and lung populations were analyzed by FACS.

### Statistics

The statistical significance of differences between conditions and treatments was analyzed with GraphPad Prism. The statistical tests used were the Student *t* test and one- or two-way ANOVA. For experiments with the LY-411575 inhibitor, statistical analysis included Tukey’s multiple comparison test. In all graphs, data are presented as mean ± SEM. Regardless of the test applied, differences were considered statistically significant at P ≤ 0.05. Specific tests and statistical values are indicated in each figure legend.

### Online supplemental material

Fig. S1 shows additional data relevant to Fig. 1. Fig. S2 shows a comparative transcriptomic analysis of bulk RNA-seq and public array data. Fig. S3 provides a comprehensive ATAC-seq analysis and genome-wide binding of RXR signaling for AM function, identity, and chromatin regulation. Fig. S4 shows cell-to-cell communication analysis and provides *in vitro* and *in vivo* evidence that DLL4 signaling is necessary for AM maturation and differentiation. Fig. S5 shows RBPJ, RXR, SMAD, and STAT5 cooperate in the regulation of AM-core gene expression. Table S1 lists the deregulated genes in *Cd11c*^*Cre+*^*Rxrab*^*fl/fl*^ versus *Rxrab*^*fl/fl*^ AM ([FC] ≤ −1.5, p-adj ≤0.1). Table S2 shows the closed peaks in *Cd11c*^*Cre+*^*Rxrab*^*fl/fl*^ versus *Rxrab*^*fl/fl*^ mAMs (log2[FC] ≥1, [FDR] ≤0.05). Table S3 shows lists of RXRa peaks in *Cd11c*^*Cre+*^*Rxrab*^*fl/fl*^ versus *Rxrab*^*fl/fl*^ mAMs ([FDR] ≤0.05). FOLD values are calculated as log2(KO/WT), and therefore negative values represent higher ocupation in WT. Table S4 shows the H3K27ac HT-ChIPmentation peaks in WT mAMs. Table S5 shows PPARγ-binding peaks in WT mAMs ([FDR] ≤0.05). Table S6 shows SMAD4 binding peaks in WT mAMs ([FDR] ≤0.05). Table S7 shows RBPJ binding peaks in WT mAMs ([FDR] ≤0.05). Table S8 indicates the primers used for mouse genotyping, qPCR experiments, and indexed library preparation for ATAC-seq and HT-ChIPmentation. Table S9 indicates all the antibodies used in this study. Table S10 provides detailed sequencing quality metrics.

## Supplementary Material

Table S1 lists the deregulated genes in *Cd11c*^*Cre+*^*Rxrab*^*fl/fl*^ versus *Rxrab*^*fl/fl*^ AM ([FC] ≤ −1.5, p-adj ≤0.1).

Table S2 shows the closed peaks in *Cd11c*^*Cre+*^*Rxrab*^*fl/fl*^ versus *Rxrab*^*fl/fl*^ mAMs (log2[FC] ≥1, [FDR] ≤0.05).

Table S3 shows lists of RXRa peaks in *Cd11c*^*Cre+*^*Rxrab*^*fl/fl*^ versus *Rxrab*^*fl/fl*^ mAMs ([FDR] ≤0.05).

Table S4 shows the H3K27ac HT-ChIPmentation peaks in WT mAMs.

Table S5 shows PPARγ-binding peaks in WT mAMs ([FDR] ≤0.05).

Table S6 shows SMAD4-binding peaks in WT mAMs ([FDR] ≤0.05).

Table S7 shows RBPJ-binding peaks in WT mAMs ([FDR] ≤0.05).

Table S8 indicates the primers used for mouse genotyping, qPCR experiments, and indexed library preparation for ATAC-seq and HT-ChIPmentation.

Table S9 indicates all the antibodies used in this study.

Table S10 provides detailed sequencing quality metrics.

SourceData F1 is the source file for Fig. 1.

## Data Availability

RNA-seq, ATAC-seq, and HT-ChIP-mentation-seq data are deposited in the National Center for Biotechnology Information Gene Expression Omnibus, with accession number GSE243165. All other data are present in the article and supplementary information.
