# Supplementary material for: A signal-responsive cooperative transcription factor network determines alveolar macrophage identity
Source: J Exp Med. 2026 Jun 2;223(7):e20242085. doi: 10.1084/jem.20242085 (PMC13228567; doi:10.1084/jem.20242085)

SourceDataF#1F

Western blot **anti-SP-A**

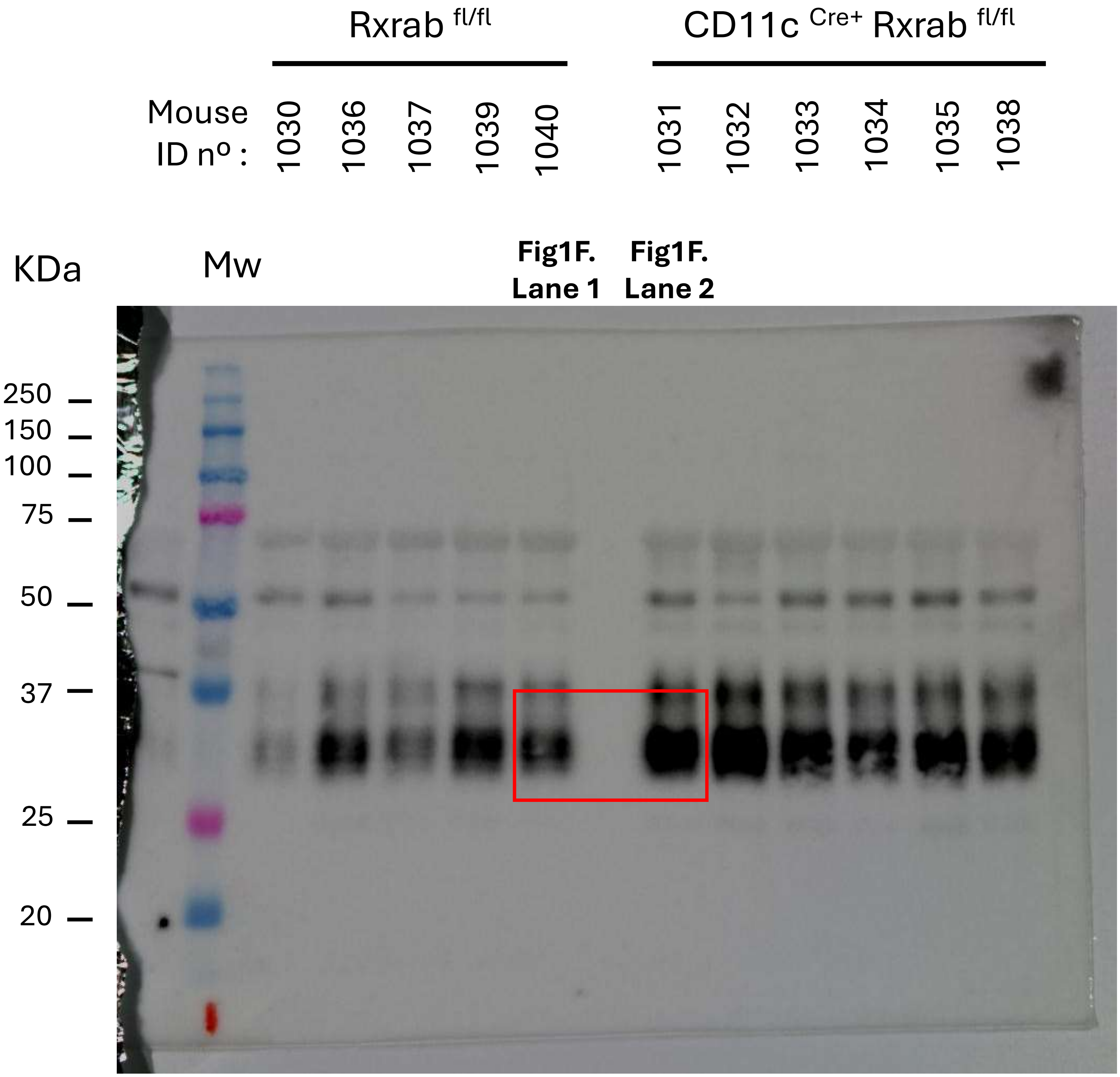

Western blot **anti-SP-B**

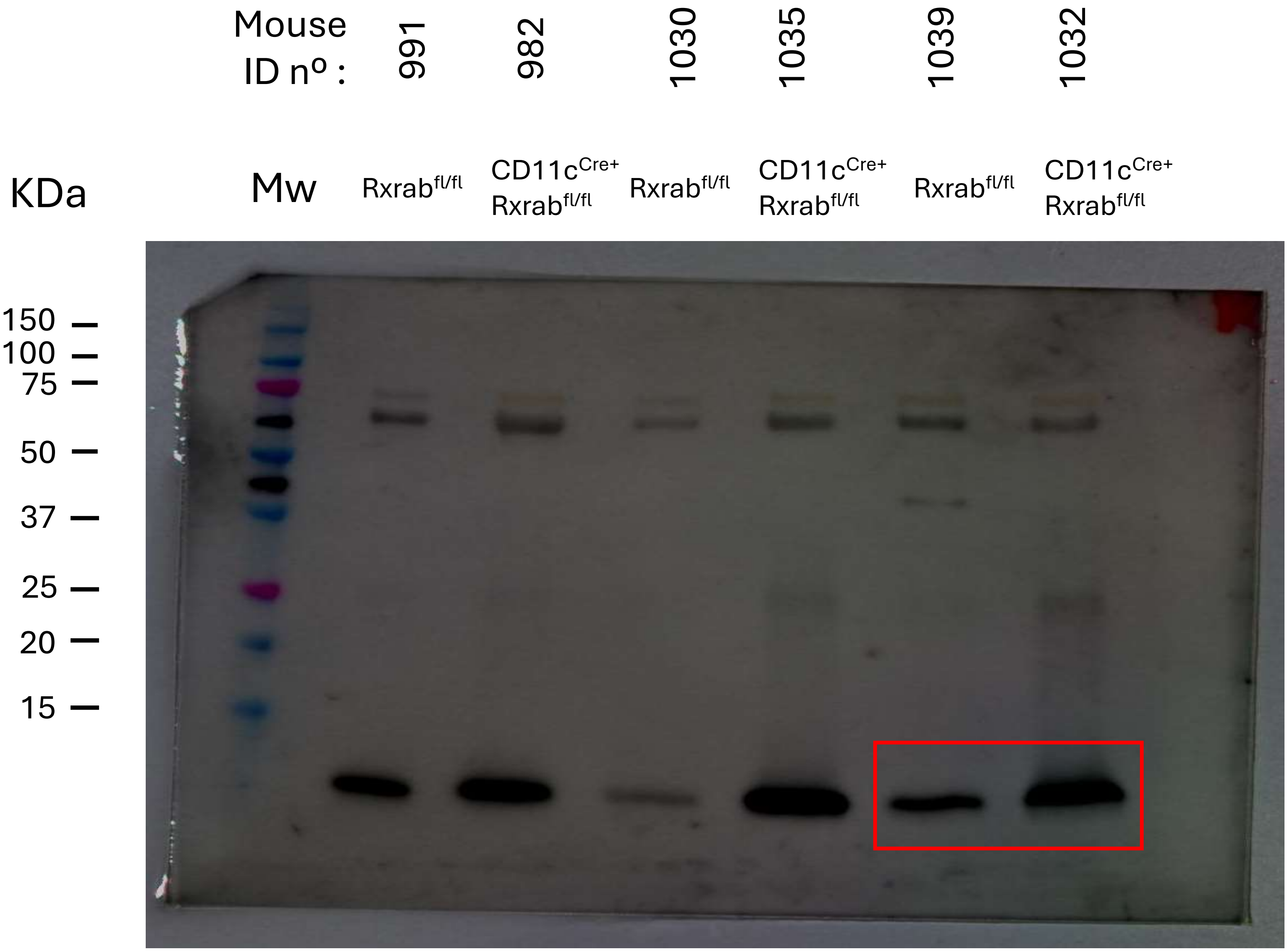

Western blot **anti-SP-C**

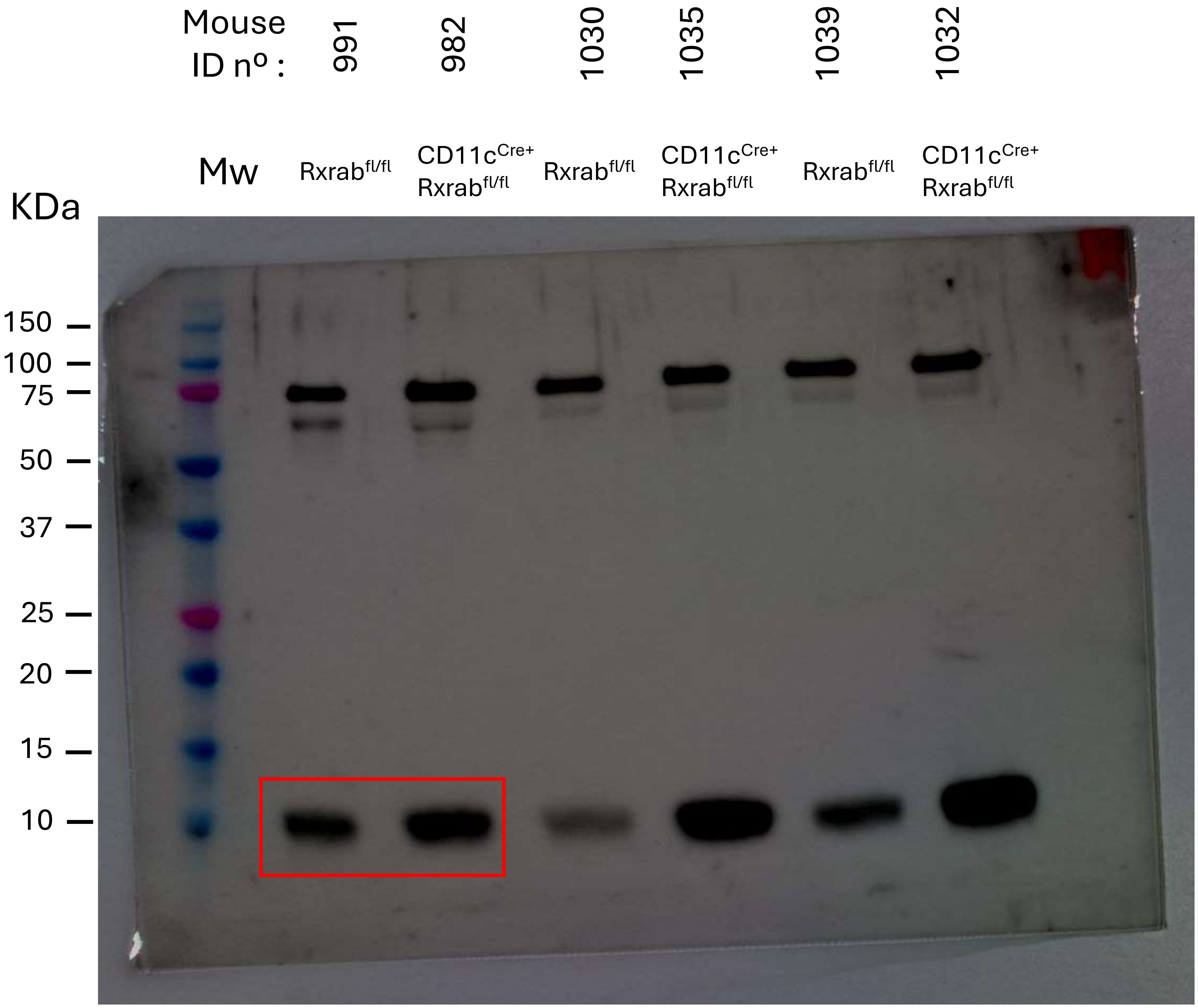

991	982	1030	1035	1039	1032
WT	KO	WT	KO	WT	KO

Western blot **anti-SP-D**

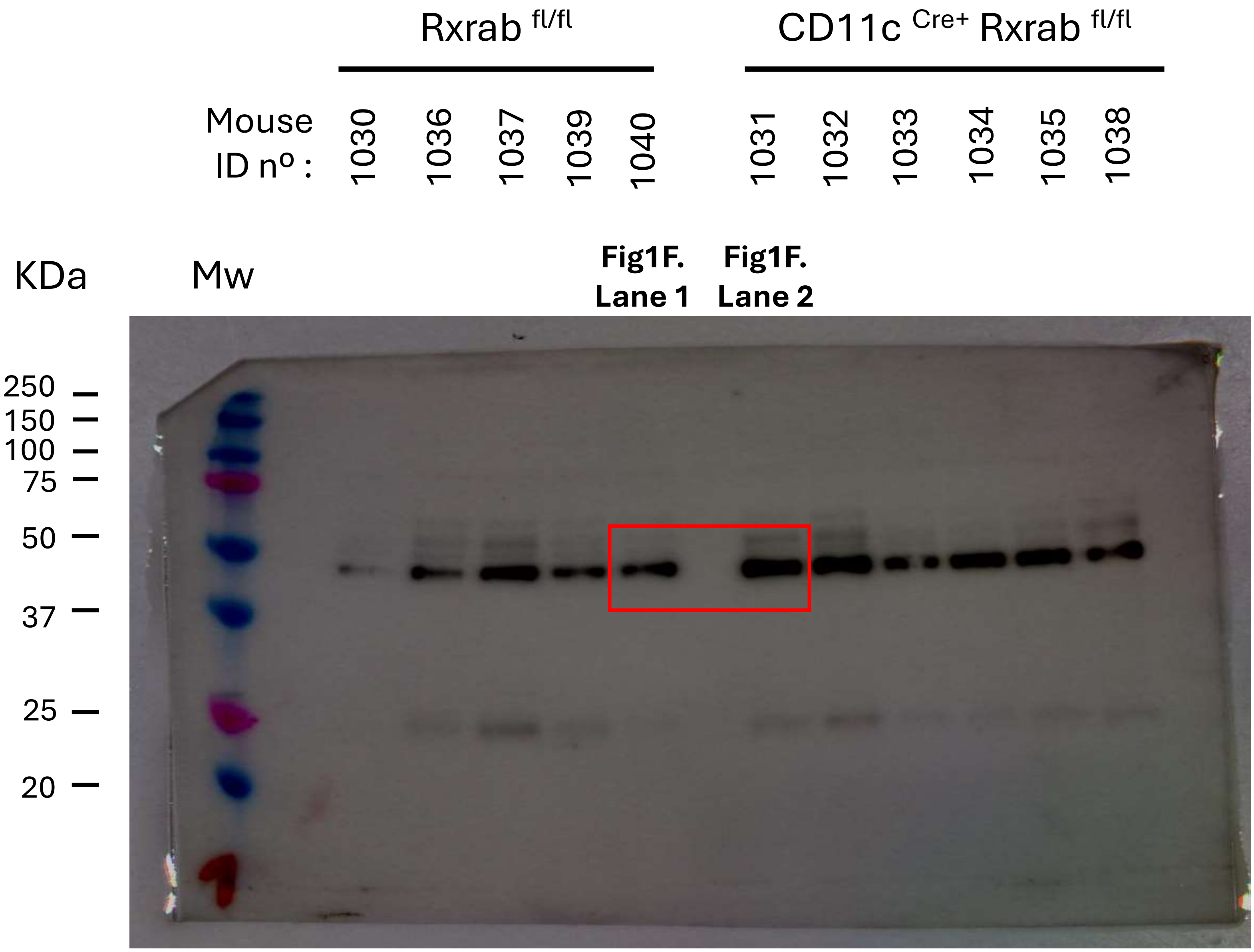

Supplement: SourceData F1 — is the source file for Fig. 1. [file jem_20242085_sourcedataf1.pdf]
